# The REN4 rheostat dynamically coordinates the apical and lateral domains of *Arabidopsis* pollen tubes

**DOI:** 10.1038/s41467-018-04838-w

**Published:** 2018-07-03

**Authors:** Hui Li, Nan Luo, Weidong Wang, Zengyu Liu, Jisheng Chen, Liangtao Zhao, Li Tan, Chunyan Wang, Yuan Qin, Chao Li, Tongda Xu, Zhenbiao Yang

**Affiliations:** 1 Shanghai Center for Plant Stress Biology and Shanghai Institute of Plant Physiology and Ecolog, Shanghai Institutes for Biological Sciences Chinese Academy of Sciences, 201602 Shanghai, China; 2 Center for Plant Cell Biology, Institute of Integrative Genome Biology, and Department of Botany and Plant Sciences, University of California, Riverside, CA 92508 USA; 3 School of Life Sciences, East China Normal University, 200241 Shanghai, China; 4 FAFU-UCR Joint Center for Horticultural Biology and Metebolomics, Institute of Science and Technology, Fujian Agriculture and Forestry University, 350002 Fuzhou, China

## Abstract

The dynamic maintenance of polar domains in the plasma membrane (PM) is critical for many fundamental processes, e.g., polar cell growth and growth guidance but remains poorly characterized. Rapid tip growth of *Arabidopsis* pollen tubes requires dynamic distribution of active ROP1 GTPase to the apical domain. Here, we show that clathrin-mediated endocytosis (CME) coordinates lateral REN4 with apical ROP1 signaling. REN4 interacted with but antagonized active ROP1. REN4 also interacts and co-localizes with CME components, but exhibits an opposite role to CME, which removes both REN4 and active ROP1 from the PM. Mathematical modeling shows that REN4 restrains the spatial distribution of active ROP1 and is important for the robustness of polarity control. Hence our results indicate that REN4 acts as a spatiotemporal rheostat by interacting with ROP1 to initiate their removal from the PM by CME, thereby coordinating a dynamic demarcation between apical and lateral domains during rapid tip growth.

## Abstract (web summary)

Polar growth of pollen tubes is established by polar localization of ROP1 GTPase activity at the apical tip. Here, Li et al. identify REN4 as a ROP1 interaction partner that restrains the ROP1 distribution to the apex by promoting ROP1 endocytosis from lateral domains of the pollen tube membrane.

## Introduction

Cell polarity is a fundamental cellular feature essential for development, growth, and survival of various organisms, and requires the spatial restriction of cellular signaling that relies on the asymmetric distribution of signaling proteins^1–6^. Much of our knowledge on the mechanisms underlying the asymmetric distribution of polarity components has come from the studies of cell polarity in *Caenorhabditis elegans* zygotes and yeast cells, where the sites of apical–basal polarity are relatively stable after polarity establishment^6,7^. In these systems, local positive feedback signaling is required for the establishment of the apical–basal polarity, while mutual local inhibition is important for the polarity maintenance^8–11^. In migrating cells and tip-growing cells, such as pollen tubes in plants, fungal hyphae, and animal neuronal axons, the polar signaling machinery that defines the moving front or the growing apical domain of these cells is highly dynamic, due to their rapid displacement during polar cell growth or movement. In response to external guidance signals, the apical signaling machinery exhibits dynamic redistribution to steer growth or movement in a new direction. The mechanisms for the dynamic spatiotemporal maintenance of the growing or moving fronts in these cells have yet to be elucidated.

The pollen tube is an essential structure for plant sexual reproduction and also serves an excellent model for the study of the spatiotemporal control of the dynamic polar domains^1,12–16^. Pollen tubes undergo rapid and guided tip growth through multiple female tissues into the ovule to deliver sperms for fertilization. In vitro cultured pollen tubes elongate uni-directionally to form a tubular cell via a self-organizing mechanism for generating and maintaining the apical growing domain of the plasma membrane (PM)^12,17,18^. The apically localized Rho-family small GTPases, ROP1 (AT3G31500), is a key regulator in this self-organizing mechanism^1,12,18–28^. The PM distribution of active ROP1 defines the apical growing domain of pollen tubes and regulates polar exocytosis^18,19,23,24,26,29^. Modeling and experimental studies suggest that exocytosis is central to the self-organizing generation and maintenance of the apical ROP1 GTPase by feed forward and feedback regulations^18^. The exocytosis-dependent feed forward is required for the establishment of the apical ROP1 polarity. The exocytosis-mediated apical targeting of the REN1 RhoGAP participates in the negative feedback to limit ROP1 signaling to the apical domain, as knocking out REN1 caused dramatic expansion of the apical ROP1 domain and growth depolarization^23^. REN1 acts as a global inhibitor of the apical ROP1 activation^18,23,25^, in contrast to the local/lateral inhibition found in yeast cells and *C*. *elegan* zygotes^8,30,31^. Such a global inhibition by a negative regulator of the apical polarity appears to be common for fast tip growing cells, as Cdc42 GAP has a similar role in maintaining cell polarity in fungal hyphae^32,33^.

The apical polarity signaling machinery needs to be separated from but dynamically coordinated with the lateral domain during fast tip growth. In pollen tubes, there exists a collar or shoulder domain dynamically flanking the apical and lateral domains^12–14,34^. The collar domain is characterized by the distribution of PIP2, cortical actin ring (collar), and components for clathrin-mediated endocytosis (CME)^23,34–39^. CME has been proposed to be active in the collar domain and appears to be critical for pollen tube growth and polarity maintenance^37,38,40,41^. Interestingly, the analogous Rho GTPase (CDC42)-based apical domain and endocytosis-marked collar domain also exist in growing fungal hyphae^42,43^. However, in both pollen tubes and fungal hyphae, how the collar domain is generated and dynamically maintained, what roles it plays, and how the collar domain coordinates with the apical domain and lateral domains remain enigmatic.

In this study, we identify a WD40 domain protein REN4 (*ROP1* *enhancer* *4*, AT2G26490) dynamically localized in the lateral and collar/apical domains of tip growing pollen tubes and plays a critical role in the precise spatiotemporal coordination between the apical and lateral domains by integrating the apical ROP1 signaling system with the collar/apical CME pathway. Consequently, REN4 acts as a rheostat for the regulation of both polarity and growth rate during pollen tube growth. Thus, our findings uncovered a novel mechanism underlying the coordination between the front and back polarities in a spatiotemporally dynamic polar system.

## Results

### *REN4* regulates pollen tube polarity and growth

**Fig. 1:**
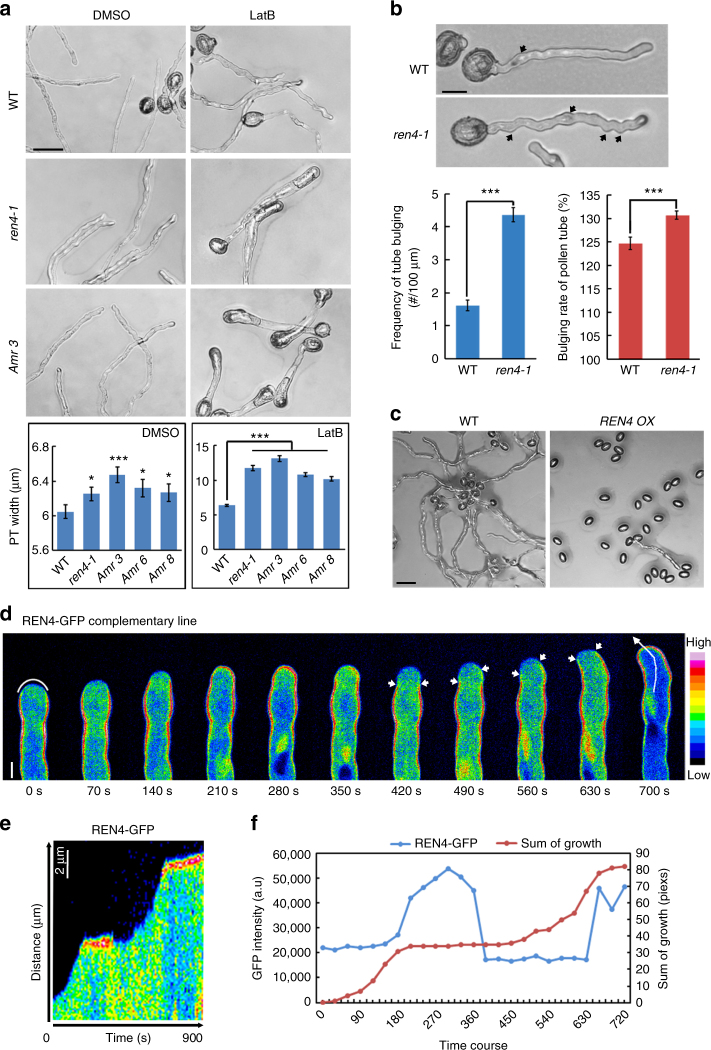
REN4 participates in pollen tube tip growth in *Arabidopsis*. **a** Pollen tube phenotypes and width quantitative analysis of wild type (WT), *ren4-1* and *REN4* artificial microRNA (*Amr*) lines. The pollen was incubated for 4 h on the medium containing either DMSO or l nM Latrunculin B (LatB). Scale bar = 50 µm. Data are represented as mean ± SEM, *n* > 63 from three biological replicates. *p < 0.05 (one-tailed unpaired *T*-test), ***p < 0.001 (one-way ANOVA, Turkey test). **b** The image and quantitative analysis of wavy (nodulating) pollen tube shape in WT and *ren4-1*. Bulge size and frequency of pollen tubes cultivated for 1 h on medium were measured. Data are represented as mean ± SD, *n* ≥ 48 from three biological replicates. Scale bar = 30 µm. ***p < 0.001 (two-tailed unpaired *T*-test). **c** The pollen tube of WT and *REN4* overexpression line (*REN4 OX*) after 5 h cultivation on medium. *REN4pro::REN4* expression in WT inhibited pollen tube germination and growth. Scale bar = 50 µm. **d** A representative time series of *REN4pro:REN4-GFP* taken from the movie shown in Supplementary Movie 1 (*n* = 90 frames, *t* = 900 s). Images shown are in pseudo-colors. Arrows indicate the REN4-GFP exclusion domain in the apical PM during pollen tube growth re-orientation (note the asymmetric distribution of the REN4-exclusion domain started at 420 s). Scale bar = 5 µm. See also Supplementary Movie 1. **e** Kymograph analysis of corresponding line (700 s image in **e**, which is along the tip center of the growing pollen tube shown in **d**. Scale bar = 2 µm. See also Supplementary Movie 1. **f** Correlation between net growth and REN4-GFP intensity at the apical PM in pollen tube. The sum of tip growth was calculated by net advance of the cell tip margin between two consecutive images. REN4-GFP intensity is the average value of 9.5 µm apical PM using Image J. (The region example is shown in 0 s image of **e**)

To dissect the mechanisms for the regulation of the apical ROP1 activity, we developed a novel screen for mutants with growth depolarization in *Arabidopsis* pollen tubes that is sensitized by a sub-inhibitory dosage of Latrunculin B (LatB) (Supplementary Fig. 1a), which promotes the depolymerization of actin microfilaments and enhances the phenotype of ROP1 over-activation mutants, such as *ren1-3*^22,23,44^. The synergistic action of LatB with *ren1-3* was explained by the requirement of actin microfilament-dependent REN1 (AT4G24580) targeting to the pollen tube tip for the function of REN1 to inactivate ROP1^22,23,44^. Thus LatB-sensitized growth depolarization mutants were expected to show enhanced ROP1 activation in pollen tubes. From 3000 homozygous T-DNA insertional mutant lines (CS27941, CS27942, and CS27943) available in ABRC, we isolated a series of mutants whose pollen tubes exhibited wider tips (reduced growth polarity) in the presence of 1 nM LatB, which had little effect on the polar growth of wild type *Arabidopsis* pollen tubes except making them wavier (Fig. 1a)^45,46^. One of these mutants, *ren4-1* (Salk_018094C) was further analyzed in this study. Treatment with 1 nM LatB greatly increased the width of *ren4-1* pollen tubes (Fig. 1a). In the absence of LatB, *ren4-1* pollen tubes were slightly wider (6.25 ± 0.08 µm, *n* = 3 biological replicates) compared to wild type tubes (6.05 ± 0.08 µm, *n* = 3 biological replicates) (Fig. 1a), but were much wavier (Fig. 1b). The *ren4-1* mutant exhibited greatly enhanced pollen germination rates and longer tubes after 1 h germination (Supplementary Fig. 1c). However, the rate of elongation in *ren4-1* pollen tubes was gradually reduced after 2 h germination (Supplementary Fig. 1c). These *ren4-1* phenotypes mimic those induced by weak ROP1 overexpression^23,25,47^. These observations suggest that REN4 (AT2G26490) participates in the spatiotemporal control of pollen tube tip growth likely by interacting with an actin-dependent pathway to regulate ROP1 signaling.

The *ren4-1* mutant contained a T-DNA insertion in the 460th codon of the only exon of At2g26490 (referred to as *REN4* in this study) and showed dramatic reduction in its transcription and translation (Supplementary Fig. 1b, d, e). *REN4* encodes a 465-aa protein with seven WD40-repeats motif (Supplementary Fig. 1b), and is identical to *JINGUBANG* recently found to inhibit pollen germination in moist environments^48^. Microarray data, the *REN4p::GUS* expression and RT-PCR assay showed that *REN4* is expressed in mature pollen and pollen tubes (http://pollen.umd.edu/)^48–50^ (Supplementary Fig. 3a–c). Consistently, downregulation of *REN4* by pollen-specific expression of *REN4* artificial microRNAs (amiRNAs) caused *ren4-*like pollen tube phenotypes (Fig.1a, Supplementary Fig. 1c, d). On the contrary, *REN4* overexpression using *REN4p::REN4* or *REN4p:REN4-GFP* construct in wild type background inhibited pollen germination and tube elongation (Fig.1c), which was similar to the report of JINGUBANG^48^. Introduction of a full-length genomic DNA sequence of *REN4* rescued all of the phenotypes of *ren4-1* (Supplementary Fig. 2)^48^. Taken together, these results confirm a role for *REN4/JINGUBANG* in regulating the tip growth and polarity of pollen tubes.

### The PM distribution of REN4 is linked to pollen tube growth

To assess how REN4 spatiotemporally regulates pollen tube tip growth, its dynamic subcellular distribution was investigated in the complementation line, *REN4p::REN4-GFP ren4-1* (Supplementary Fig. 2b). As previously reported^48^, the REN4-GFP fusion protein was distributed in the vegetative nuclei and the cytosol in pollen grains and pollen tubes (Supplementary Fig. 3d). Furthermore, we found that REN4-GFP was also clearly localized to the PM (Supplementary Fig. 3d), although this REN4-GFP distribution pattern was not examined by Ju et al.^48^. The PM distribution was more concentrated in the PM region of the emerging tube and particularly in the region flanking the emerging tube. In elongating tubes, the PM distribution of REN4-GFP persisted (Supplementary Fig. 3d). The PM localization of REN4-GFP was confirmed by co-staining with membrane lipophilic styryl dye FM4-64 (Supplementary Fig. 3e). Snapshot confocal imaging showed that in the majority of pollen tubes after 3 h cultivation in vitro (72%:106 out of 143 tubes), REN4-GFP was found in the lateral PM region and excluded from the apical PM domain (Fig. 1d), where active ROP1 is distributed^25,51^. Time lapse imaging of *REN4p::REN4-GFP ren4-1* pollen tubes showed that REN4-GFP was periodically localize to the apical PM as well, and the apical distribution was associated with the cessation of pollen tube elongation, whereas its exclusion from the apical PM was immediately followed by rapid growth (Fig.1d–f and Supplementary Movie 1). Furthermore, in pollen tubes undergoing reorientation, the REN4-GFP excluded region of the PM predicts the new growth direction (Fig.1d (490s–630s)). These observations indicate the REN4 distribution to the apical PM is closely associated with pollen tube growth inhibition. FRAP analysis showed that REN4-GFP moved laterally along the PM (Supplementary Fig. 3f, g), suggesting that its dynamic distribution to the apical PM most likely takes place by lateral diffusion.

### REN4 antagonizes ROP1 signaling in the apical PM domain

**Fig. 2:**
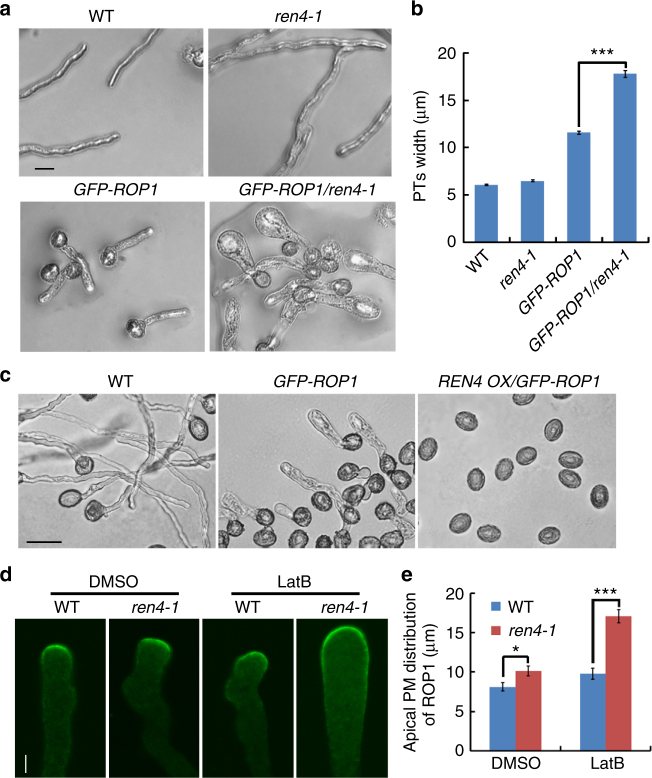
REN4 antagonizes the activity and function of ROP1 in pollen tube. **a**, **b** Growth depolarization in *GFP-ROP1* overexpressing pollen tubes was enhanced by the *ren4-1* mutation. The representative pollen tube images in **a**. The quantitative analysis of pollen tube width in **b**. Scale bar = 20 µm. *n* ≥ 58 from three biological replicates. **c** The pollen tube of WT, *GFP-ROP1*, and *REN4-OX/GFP-ROP1*. REN4 inhibited the pollen tube germination and growth of *GFP-ROP1* overexpression line in *Arabidopsis*. Scale bar = 50 µm. **d**, **e** The ROP1 distribution was expanded in the apical PM of *ren4-1* pollen tube and this expansion was greatly enhanced by treatment with LatB. The ROP1 was detected by immunostaining using ROP1 antibody. The representative images were shown in **d**. The quantitative analysis of ROP1 distribution was shown in **e**. Scale bar = 5 µm. Representative result of three independent experiments is presented, *n* ≥ 10. In **b** and **e**, data are represented as mean ± SEM, *p < 0.05 (one-tailed unpaired *T*-test), ***p < 0.001 (two-tailed unpaired *T*-test)

The *ren4-1* phenotypes and the subcellular distribution of REN4 imply a probable link between REN4 and the ROP1 signaling. To assess this hypothesis, we first crossed *ren4-1* and REN4 overexpression line with the line that moderately overexpressed GFP-ROP1, which exhibited a weak growth depolarization in pollen tubes^19,47^. As shown in Fig. 2a–c, the growth depolarization of the GFP-ROP1 pollen tubes was greatly enhanced by *ren4-1*. We previously showed that ROP1 overexpression or activation not only caused growth depolarization in pollen tubes, but also induced early germination and increased germination rates^23,47^. Interestingly, REN4 overexpression nullified pollen germination in ROP1 overexpressing tubes. These genetic analyses suggest that *REN4* functionally antagonizes ROP1 signaling in the regulation of *Arabidopsis* pollen tube tip growth. Based on the enhancement of growth depolarization of GFP-ROP1 pollen tubes by *ren4-1* and the exclusion of REN4 from the growing domain containing active ROP1 (Figs. 1d and 2a), we hypothesized that REN4 restricts the lateral expansion of active ROP1 from the apical PM region. To test this, we compared the distribution of ROP1 in the apical PM between wild type and *ren4-1* pollen tubes immuno-stained with anti-ROP1 antibodies^52^. Under normal growth conditions, ROP1 distribution to the apical PM in *ren4-1* tubes was slightly wider compared to that in wild type tubes (Fig. 2d, e). In the presence of 1 nM LatB, however, the ROP1 distribution to the apical PM was greatly expanded into the subapical PM of *ren4-1* pollen tubes (Fig. 2d, e). These results suggest that REN4 and an actin-mediated pathway act synergistically to regulate ROP1 distribution in the apical PM.

### REN4 is spatiotemporally associated with active ROP1

**Fig. 3:**
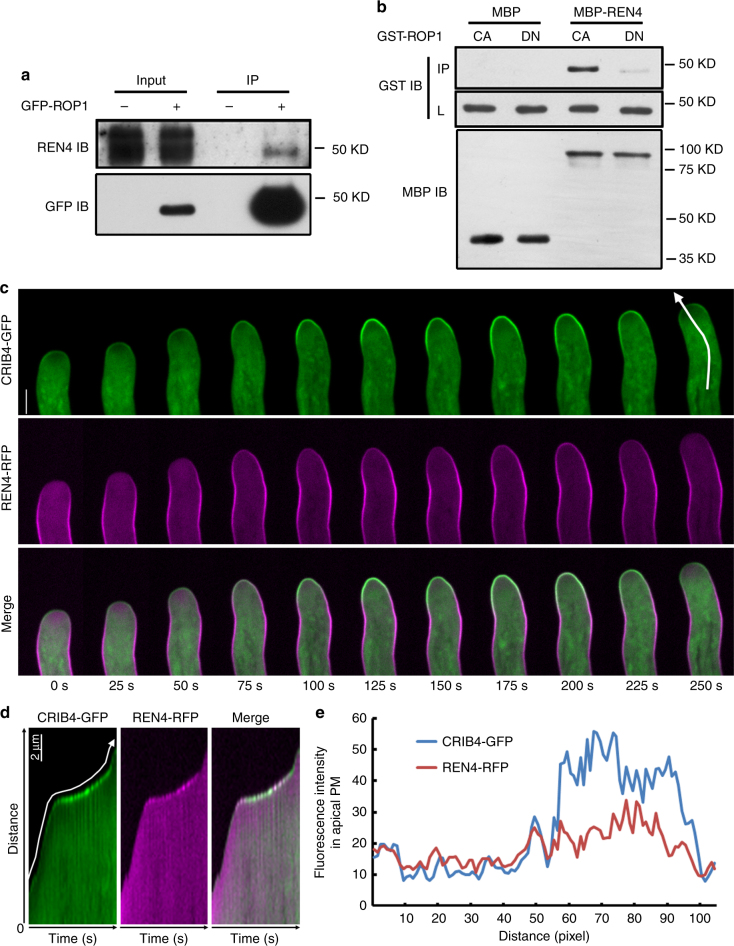
The interaction between REN4 and active ROP1 in the pollen tube. **a** The co-immunoprecipitated analysis of the interaction between ROP1 and REN4 in *Arabidopsis* pollen tube. Endogenous REN4 was detected in the protein complex precipitated from pollen tubes expressing GFP-ROP1 by GFP antibody, but not in wild type control. IP immunoprecipitation, IB Immuno-Blotting. **b** In vitro pull down assay showing that REN4 preferentially interacted with the GTP-bound active ROP1. L loading control, IP pulldown. DN, GDP-bound dominant negative form of ROP1. CA, GTP-bound constitutively active form of ROP1. **c** Spatiotemporally dynamic distribution of active ROP1 and REN4 to the apical PM of *Arabidopsis* pollen tube. The CRIB4-GFP/REN4-RFP was monitored in the semi-in vitro pollen tube of corresponding transgenic line using spinning disk confocal (*n* = 260 frames, *t* = 260 s). Scale bar = 5 µm. See also Supplementary Movie 2. **d**, **e** CRIB4-GFP/REN4-RFP kymograph from the corresponding line shown in **c**, which is along the apical center of the growing pollen tube (Supplementary Movie 2, *n* = 50 frames, *t* = 250 s), and corresponding fluorescence intensity profiles at the apical PM (**e**). Scale bar = 2 µm. The line profile shows that a dramatic increase in CRIB4-GFP in the apical PM was followed by an increase in REN4-RFP and then a simultaneous reduction of both CRIB4-GFP and REN4-RFP in the apical PM

We next sought to understand the mechanism by which REN4 antagonizes ROP1 signaling. Co-immunoprecipitation (Co-IP) in the GFP-ROP1 line using REN4 native antibody showed that ROP1 and REN4 are physically associated with each other (Fig. 3a). In vitro pull down assays using *Escherichia* *coli* expressed proteins demonstrated that REN4 tagged with maltose-binding protein (MBP) directly and preferentially interacted with the GTP-bound active ROP1 compared to the GDP-bound inactive form of ROP1 (Fig. 3b).

Given the preferential interaction of REN4 with the active ROP1, we propose either or both of the following mechanisms for the suppression of ROP1 function by REN4: (1) REN4 directly inhibits the activity of ROP1, (2) REN4 regulates the distribution of active ROP1 by removing it from the apical PM. The latter is supported by the REN4 distribution on the lateral PM of growing pollen tubes and its role in restricting ROP1 to the apical PM (Figs. 1e and 2d). By interacting and removing active ROP1 from the PM, the laterally localized REN4 would restrict the expansion of active ROP1 from the apical PM region. We visualized active ROP1 using CRIB4-GFP^18^. Time lapse analysis showed that during oscillatory growth, dynamic and relatively weak ROP1 activity at the apical PM coincided with fast rates of pollen tube elongation, while persistent high levels of active ROP1 at the tip are associated with slower growth (Supplementary Fig. 4a). The overall CRIB4-GFP pattern appeared to be similar to the correlation between pollen tube growth and the distribution of REN4 to the tip (Fig.1f). However, detailed time-lapse imaging and kymograph analyses showed that when the apical active ROP1 (PM-localized CRIB4-GFP) increased and spread laterally, REN4-RFP first encountered active ROP1 in the collar region (the yellow signal showed the overlapping distribution here) (frames 100–150 s, Fig. 3c, d). As the apical active ROP1 continued to increase, REN4-RFP spread to the extreme apex. The overlapping distribution of active ROP1 and REN4 was then followed by the rapid removal of REN4 and active ROP1 from the PM (Fig. 3c, d). Then some low level of CRIB4-GFP remained in the apical PM, but REN4-GFP completely disappeared there (Fig. 3c). During growth reorientation, an asymmetric increase and then decrease in CRIB4-GFP and REN4-RFP intensity in the PM region was observed at the new growth site (Fig. 3c and Supplementary Movie 2). During the rapid removal of both REN4-RFP and CRIB4-GFP, the cytoplasmic REN4-RFP increased dramatically (Fig. 3c, d), suggesting that REN4-RFP may be actively internalized from the PM. In tobacco pollen tubes transiently co-expressing GFP-ROP1 and RFP-REN4, we observed a dramatic decrease in the fluorescence intensity ratio between the PM and the cytosol for both fusion proteins in the pollen tubes, compared with tubes expressing either one of the genes (Supplementary Fig. 4e–g). On the basis of these results, together with the physical and functional interaction between active ROP1 and REN4, we propose that REN4 antagonizes ROP1 signaling by promoting the removal of active ROP1 from the PM after ROP1 and REN4 interacts with each other.

This hypothesis is supported by the changes in the distribution of ROP1 protein (Fig. 2d, e) and active ROP1 in *ren4-1* pollen tubes (Supplementary Fig. 4b–d). The active ROP1 of *ren4-1* tubes was accumulated to the apical PM as in wild type tubes, but spread to a wider region of the tip compared to that in wild type tubes (Supplementary Fig. 4b, c). Furthermore, the active ROP1 distribution to the apical PM of *ren4-1* tubes persisted longer compared to wild type tubes, resulting in less dynamic changes in the apical ROP1 activity and increased lengths of oscillation periods (Supplementary Fig. 4d). These changes were associated with slower pollen tube elongation (Supplementary Fig. 1c). These findings are consistent with our previous observations that ROP1 activation is required for pollen tube growth, but high growth rates are associated with dynamic changes in the apical ROP1 activity whereas persistent high levels of ROP1 activity are associated with slower growth^19,25,26,51^.

### REN4 interacts with EAP1 for CME

**Fig. 4:**
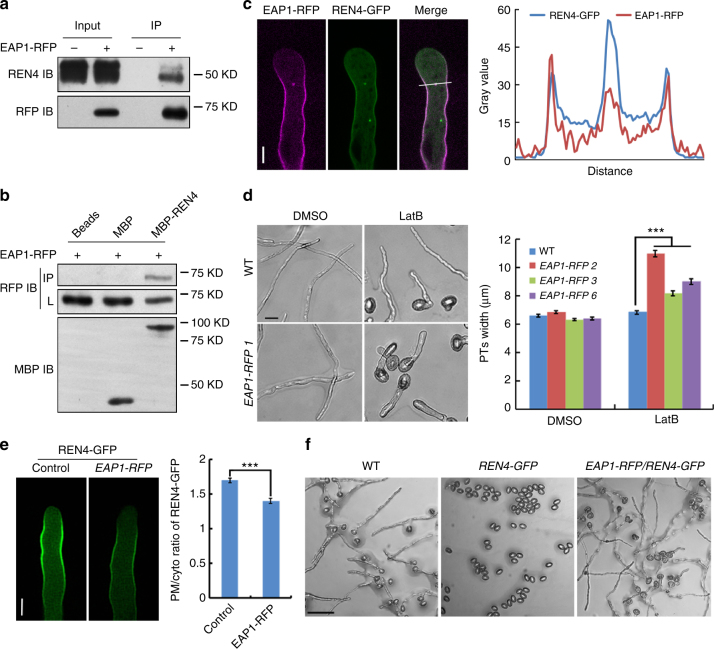
EAP1 counteracts REN4 in pollen tube growth in *Arabidopsis*. **a** EAP1 co-immunoprecipitated with REN4 in *Arabidopsis* pollen tube. Endogenous REN4 was detected in the EAP1-RFP precipitate from *Arabidopsis* pollen tubes expressing *EAP1pro::EAP1-RFP*. **b** EAP1-RFP from *Arabidopsis* EAP1-RFP transgenic pollen tube was pull down by MBP-REN4. MBP and Amylose beads served as the negative control. **c** The colocalization of REN4 and EAP1 in the lateral PM of *Arabidopsis* pollen tubes. Left, representative images of tubes co-expressed EAP1-RFP and REN4-GFP. Right, corresponding line plot. Scale bar = 5 µm. **d** EAP1-RFP overexpressing pollen tubes exhibited depolarized growth in the presence of 1 nM LatB. Left, representative images of pollen tubes. Scale bar = 20 µm. Right, quantitative analysis of pollen tube width. The pollen tubes were cultivated for 3 h on DMSO and l nM LatB medium. *n* ≥ 62 from three biological replicates. ***p < 0.001(one-way ANOVA, Turkey test). **e** The PM distribution of REN4-GFP was reduced by *EAP1pro::EAP1-RFP* expression. Left, Representative REN4-GFP images in control (WT) pollen tubes and those co-expressed with EAP1-RFP. Right, quantitative analysis of the REN4-GFP PM/cytosol intensity ratio. Scale bar = 5 µm. Representative result of three independent experiments is presented, *n* ≥ 52, ***p < 0.001 (two-tailed unpaired *T*-test). **f** Pollen tube germination and growth inhibition in the *REN4*-GFP expressing line was released by *EAP1-RFP* expression in *Arabidopsis*. Scale bar = 100 µm. In **c** and **e**, data are represented as mean ± SEM

To elucidate the mechanism by which REN4 regulates ROP1, we sought to identify REN4-interacting proteins. We reasoned that the functional specificity of WD40 proteins lies in the variable regions outside of the conserved WD40 repeats^53^, and thus used the N-terminal region of REN4 as bait for Y2H screening from an *Arabidopsis* flower cDNA library. Among 12 candidate interactors, an AP180 N-Terminal Homology (ANTH) family member, designated as Endocytosis Adaptor of Pollen Tube (EAP1, AT1G68110), was chosen for further analysis (Supplementary Fig. 5a). ANTH family members are known as accessary cargo adaptor for CME^54,55^. The first characterized plant ANTH protein, *Arabidopsis* AP180 (AT1G05020), is localized in the collar PM domain of pollen tubes and may be involved in the PI(4,5)P2-regulated membrane invagination during CME^37,56^. The phylogenetic analysis of ANTH family proteins in *Arabidopsis* shows that EAP1 is classified with other four members into the A1 clade of the ANTH family whose function is unclear in plants^54^. The physical interaction between REN4 and EAP1 was confirmed by Co-IP using proteins from *Arabidopsis* pollen tubes and by in vitro pull down assays (Fig.4a, b). EAP1 also interacted with Clatrhin Light Chain 1 (CLC1, AT2G02760) subunit of clathrin triskelion in pollen tubes (Supplementary Fig. 5b)^57^.

In transgenic *EAP1p::EAP1-RFP* lines, EAP1-RFP was mainly distributed along the lateral and collar regions of the PM and often excluded from the apical PM in pollen tubes (Supplementary Fig. 5c). EAP1-RFP was co-localized with REN4-GFP in the PM and vesicle-like organelles (Fig. 4c, Supplementary Fig. 5d). Interestingly, *EAP1p*::*EAP1-RFP* pollen tubes phenocopied *ren4-1*, as they exhibited LatB-induced growth depolarization (Fig. 4d). Importantly, *EAP1p*::*EAP1-RFP* greatly attenuated the abundance of REN4 in the pollen tube PM (Fig. 4e) and consistently recovered the pollen tube germination and growth inhibition caused by REN4 overexpression in *Arabidopsis* (Fig. 4f). These results suggest EAP1 functionally antagonizes REN4 by directly targeting and removing PM-localized REN4 via CME in *Arabidopsis* pollen tubes.

### CME regulates REN4 and ROP1 distribution and function

**Fig. 5:**
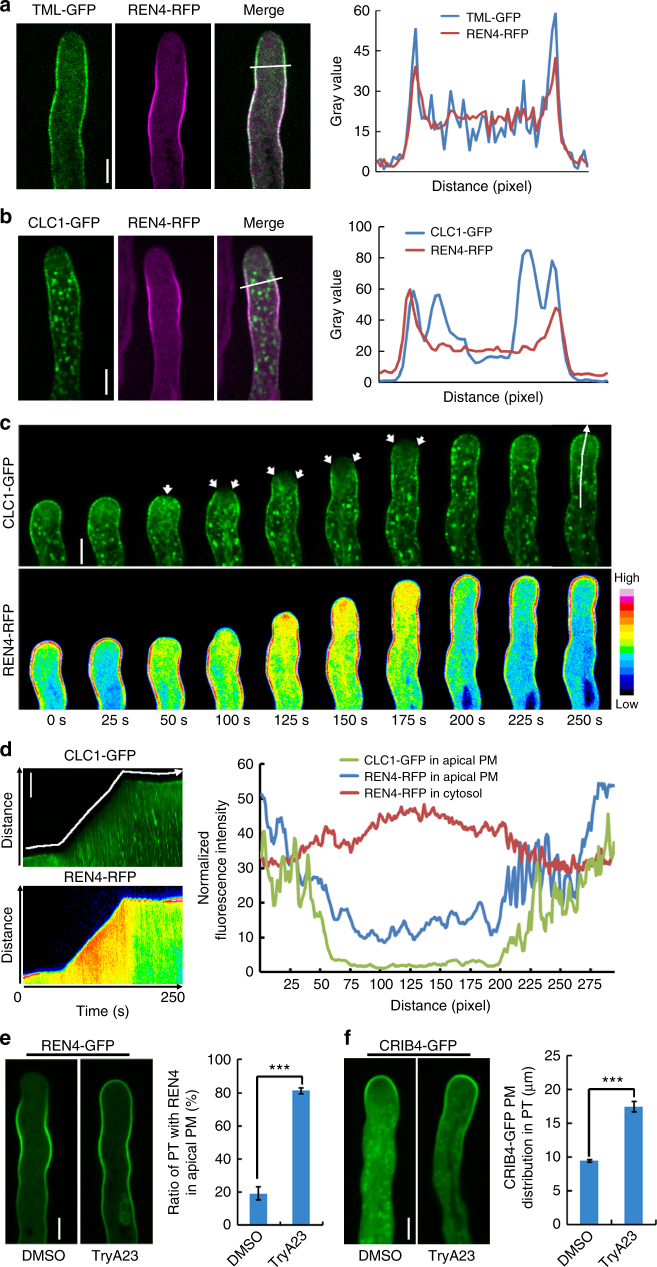
CME modulates REN4 distribution to the pollen tube apical PM. **a** REN4 and TML are colocalized to the lateral PM of *Arabidopsis* pollen tube. Left, the representative images of pollen tube co-expressing REN4-RFP and TML-GFP. Right, the intensity plot of corresponding line shown in left. Scale bar = 5 µm. **b** The distribution of REN4-RFP and CLC1-GFP in *Arabidopsis* pollen tube. Left, representative images of REN4-RFP/CLC1-GFP pollen tubes. Right, the intensity plot of corresponding line shown in left. Scale bar = 5 µm. **c** The spatiotemporal dynamic distribution of CLC1-GFP and REN4-RFP in *Arabidopsis* pollen tube. The time series from a representative movie (Supplementary Movie 3) (*n* = 250 frames, *t* = 250 s). Arrows indicate the positions where CLC1-GFP particles are rapidly moving into the cytoplasm from the PM. Scale bar = 5 µm. See also Supplementary Movie 3. **d** The kymographs (left) of REN4-RFP and CLC1-GFP along the line shown in **c**, which is along the apical center of growing pollen tube. The fluorescence intensity profiles (right) showed correlation between REN4-RFP and CLC1-GFP at the apical PM and cytosol. The intensity of REN4-RFP at apical PM and in the cytoplasm exhibited contrasting distribution. The line for plot profiles is labeled in the kymograph along PM, and the line was moved 10 pixels from PM toward into cytosol to obtain the cytosolic REN4-GFP profile. **e** Inhibition of clathrin-mediated endocytosis by TyrA23 treatment increased REN4-GFP distribution to the apical PM (left), and the PT ratio with REN4-GFP in apical PM was measured (right). Scale bar = 5 µm. **f** Inhibition of CME by TyrA23 treatment expanded CRIB4-GFP distribution into the subapical PM (left), and the apical PM distribution of CRIB4-GFP in pollen tube was measured. Scale bar = 5 µm. In **e** and **f** representative result of three independent experiments is presented. Data are represented as mean ± SEM, ***p < 0.001(two-tailed unpaired *T*-test). *n* ≥ 6 for **e** and *n* ≥ 61 for **f**

Based on the direct interaction between the active ROP1 and REN4 (Fig. 3a, b) and their simultaneous removal form the PM (Fig. 3c–e), we hypothesize that the complex of REN4 and active ROP1 is internalized by CME in pollen tubes. To test this hypothesis, we first determined whether REN4 exhibited overlapping distribution with clathrin coat proteins and their accessory proteins in *Arabidopsis* pollen tubes. TML is a component of the T-Plate accessory complex for CME and was shown to localize to the lateral PM in a pattern similar to that for REN4 and EAP1^39,58,59^. As shown in Fig. 5a, TML-GFP clearly exhibited overlapping localization with REN4-RFP along the lateral PM, and was excluded from the extreme apex like REN4-GFP and EAP1-RFP in growing pollen tubes. Furthermore, TML-GFP displayed a discontinuous distribution pattern along the PM, whereas REN4-RFP distributes uniformly along the lateral PM. Similarly, CLC1 as a necessary subunit of clathrin-related machinery^57^ also showed overlapping distribution with REN4 in pollen tubes. CLC1-GFP was highly enriched in the collar PM region just behind the extreme apex, where it exhibited a strong overlapping distribution with REN4-RFP (Fig. 5b). Furthermore, CLC1-GFP displayed punctuated dots along the PM region and in the cytosol, most likely as endocytic vesicles as previously reported^39,60,61^.

Time lapse imaging analysis showed that CLC1-GFP was co-localized with REN4-RFP in the apical PM domain during slow growth phase, concomitant with the strong apical distribution of active ROP1 (Figs. 3c and 5c; Supplementary Movies 2 and 3). This was followed by the formation of CLC1 vesicles from the apical PM and the movement of these vesicles from the PM to the cytoplasm (Supplementary Fig. 6a, b and Supplementary Movie 7). Then REN4-GFP was removed from the apical PM domain and fast growth resumed. During the fast growth phase, CLC1-RFP vesicles were formed more frequently from the collar region (Supplementary Fig. 6c and Supplementary Movie 6), where active ROP1 and REN4 rapidly disappeared (Fig. 3c). The kymograph analysis of the apical PM confirmed that both CLC1-GFP and REN4-RFP were removed from the apical PM domain just prior to the resumption of the fast growth phase (Fig. 5d and Supplementary Fig. 6a, b). The disappearance of REN4-RFP from the apical PM coincided with the dramatic increase in the cytoplasmic REN4-RFP intensity and the robust CLC1-GFP dynamic in the collar region of the PM (Fig. 5c, d).

Above observations are consistent with the hypothesis that REN4 and active ROP1 are actively removed from the PM by CME when they encounter each other. We next determined whether CME regulates REN4 and active ROP1 distribution in the PM of pollen tubes. Pollen tubes expressing REN4-GFP or CRIB4-GFP were treated for 15 min with 100 μM TryA23, a commonly used inhibitor of CME^62,63^. Our observations showed that 81 ± 4.67% (*n* = 7) of TryA23-treated pollen tubes exhibited REN4 distribution to the apical PM compared to 19 ± 9.25% (*n* = 6) of control tubes treated with DMSO (Fig. 5e). The distribution of CRIB4-GFP to the apical PM region was also greatly increased by TryA23 treatment. The average width of CRIB4-GFP distribution to the apical PM increased from 9.43 ± 1.66 μm (*n* = 3 biological replicates) in mock-treated tubes to 17.46 ± 6.73 μm (*n* = 3 biological replicates) in TyrA22-treated tubes (Fig. 5f). Therefore, inhibition of CME caused the spreading of the apical active ROP1 to the lateral domains and of the lateral REN4 to the apical domain. Furthermore, several *clahtrin heavy chain 2* (*chc2*) T-DNA insertional mutants consistently showed greatly reduced pollen tube lengths (Supplementary Fig. 6d), and a *chc2* mutation induced the expansion of REN4 distribution into the apical PM, consistent with the effect of TryA23. These results further support the hypothesis that CME coordinates the distribution of both REN4 and active ROP1 by internalizing the REN4-active ROP1 complex from the pollen tube PM.

### Lateral REN4 stabilizes the orientation of PT tip growth

**Fig. 6:**
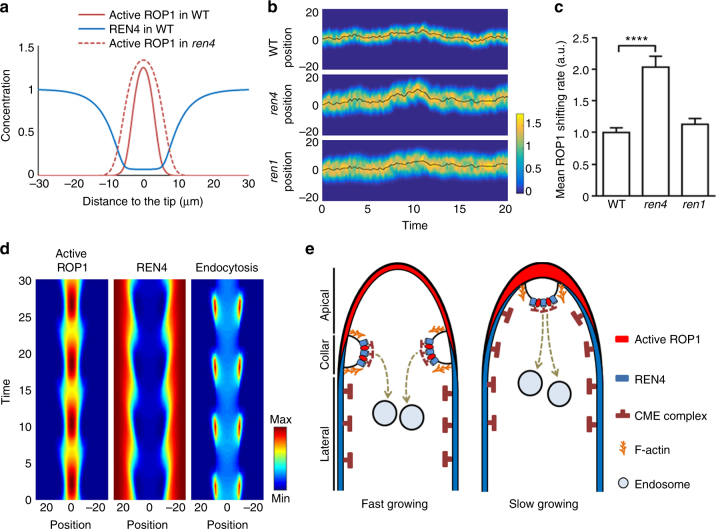
REN4 as a lateral inhibitor stabilizes the distribution of active ROP1. **a** Simulated distribution of active ROP1 and REN4 assuming that REN4 both directly inhibits ROP1 and mediates the endocytosis of ROP1. Dashed red line shows that active ROP1 expands in the absence of REN4. **b** Simulated distribution of active ROP1 over time in the presence of noise. Color shows the concentration of active ROP1. Black solid lines indicate the center of ROP1 distribution. **c** The mean shifting rate (absolute displacement per unit time) of the center of ROP1 distribution due to noises normalized by the WT value. Error bars show S.E.M. ****P ≤ 0.0001 (Kolmogorov–Smirnov test). Loss of the lateral inhibitor REN4 results in stronger disturbance of ROP1 distribution by noise, while loss of the global inhibitor REN1 does not significantly affect the robustness of ROP1 to noise. **d** The simulated dynamic processes of enodyctosis event which mediated by the interaction and distribution of active ROP1 and REN4 in the pollen tube. **e** A proposed model for clathrin-mediated endocytosis, REN4, and active ROP1 coordinated polar establishment and maintenance for the regulation of pollen tube tip growth. The interaction between REN4 and ROP1 induced CME was preferentially occurred in the collar region at fast growing stage, and the internalization site of CME is gradually moving into the apical region where active ROP1 was accumulated in and REN4 expanded into at slow growing stage

To gain further insights into the role of REN4 in the tip growth of pollen tube, we integrated the REN4-dependent CME pathway into a mathematical model for pollen tube growth we developed earlier^18^. In this model, active ROP1 polarizes at the tip of pollen tubes through exocytosis-mediated local positive feedback and RhoGAP-mediated global inhibition. Here our data suggest that REN4-mediated CME at the collar region internalizes active ROP1, preventing its invasion to the lateral domains. Since REN4 is also removed from the PM through CME, CME possibly acts as a gatekeeper, mediating the mutual inhibition between REN4 and ROP1: The joining of REN4 and active ROP1 induces local CME, leading to the removal of both. The new model developed based on these hypotheses reproduced the spontaneous formation of the complementary pattern of active ROP1 and REN4 at the tip and at the lateral domains, respectively (Fig. 6a). Removing REN4 from the system results in the mild expansion of the active ROP1 peak (Fig. 6a–c), consistent with the slightly wider distribution of ROP1 (Fig. 2d, Supplementary Fig. 4b, c) and the increased width of *ren4-1* pollen tubes with or without the presence of LatB (Fig. 1a, b). Therefore, the check-and-balance between REN4 and active ROP1 maintains the polarized distribution of ROP1 and thus regulates the growth and morphology of pollen tubes. When the time delay between the REN4-ROP1 interaction and the endocytosis of membrane proteins is considered, the model generated the spatiotemporal dynamics of REN4 and ROP1 (Fig. 6d), as experimentally observed in pollen tubes (Fig. 3c and Supplementary Movie 2). Separation of active ROP1 and REN4 to the apical and lateral regions leads to the reduction in endocyctosis. Furthermore, without lateral inhibition, ROP1 spreads to the shoulders and REN4 spreads into the tip. The overlapping of them induces endocytosis that removes both proteins from the PM, so that REN4 retracts to the flanking areas and ROP1 is maintained at the tip.

Distinct from the lateral distribution of REN4, another negative regulator of ROP1, the REN1 RhoGAP, is distributed in the apical PM and exocytic vesicles at the cell apex and therefore was considered as a global inhibitor of ROP1^23^. REN1 plays a primary role in controlling the level of ROP1 activity comparing to REN4. The *ren1* knockout pollen tubes are severely depolarized due to the extensive spreading of the apical ROP1 activity to the lateral domain^23^. In contrast, *ren4-1* pollen tubes were only mildly affected in cell width unless being sensitized by disrupting actin filaments or overexpressing ROP1. Why do pollen tubes equip with two different mechanisms for the negative regulation of ROP1 activity? The wavy phenotype of the *ren4-1* pollen tubes indicates a defect in maintaining a stable growth direction. Introduction of noises into the model results in random shifting of the position of active ROP1, which will cause changes in the orientation of growth (Supplementary Fig. 7a). Interestingly, noise-induced shifting of active ROP1 becomes much more frequent and dramatic in simulated *ren4* pollen tubes (Fig. 6b, c and Supplementary Fig. 7b–e). Decreasing the global inhibition rate (i.e. *ren1* mutant), however, has no significant impact on the stability of the ROP1 localization in the presence of noise (Fig. 6b, c and Supplementary Fig. 7b–e). Therefore, there is a division of labors between the two classes of ROP1 inhibitors: REN1 as a global inhibitor plays the major role in suppressing the magnitude of ROP1 activity to maintain the size of the growth domain, whereas REN4 as a lateral inhibitor restrains the ROP1 cap from wandering by filtering out noises and thus is important for maintaining a robust growth direction under noisy background.

## Discussion

The maintenance of polar domains requires the spatial restriction of the feed-forward activated polarity signaling machinery, and has been proposed to rely on two distinct inhibition mechanisms: (1) global down-regulation and (2) lateral inhibition^8,23,25^. In rapidly tip-growing or moving cells, a tight but dynamic coordination between the apical and basal domains is necessary and could not be easily explained by either of the two restriction mechanisms alone. Our findings here uncovered a novel mechanism for the dynamic coordination between the apical and the lateral (basal) domains in rapidly growing pollen tubes. The interaction between the apical active ROP1 GTPase and the lateral REN4 scaffolding protein induces their CME-dependent removal from the PM, generating a dynamic demarcation that separates but tightly coordinates the apical and lateral cortical domains. Moreover, the REN4-mediated and CME-mediated coordination of the apical and lateral domains is important for the stabilization of pollen tube growth direction. Similar coordination between the apical and basal domains may also be crucial for the spatiotemporal regulation of tip growth or cell migration in other systems, such as fungal hyphae and animal neuronal axons.

Our results support a new mechanism for the regulation of CME to internalize specific membrane-associated proteins. In the conventional CME, the binding of transmembrane cargos to a cytoplasmic adaptor protein initiates clathrin assembly and activates CME. REN4 as a membrane-associated cargo colocalizes with CME components in the lateral PM of pollen tubes, yet no CME is evident in this region based on CLC1-GFP imaging. CME mainly occurs in the apical domain and collar regions of the PM where REN4 encounters active ROP1. Furthermore, the removal of both REN4 and active ROP1 from the PM is dependent upon CME. Taken together our results suggest that the interaction between REN4 and active ROP1 triggers CME to remove them from the apical and collar regions of the PM. This consistent with several previous studies indicating that in growing pollen tubes CME predominantly occurs in the restricted collar region, which is enriched in PIP2, PIP2-binding CME accessory proteins (including ENTH and ANTH) and PLCs that metabolize PIP2^37,64,65^. Regulation of cargo proteins by phosphorylation for cross inhibition between polar domains has been proposed^6,8^. Our findings suggest a novel mechanism for the regulation of endocytosis that is based on the interaction between cargo proteins. The binding of active ROP1 to REN4 may alter REN4 conformation and thus enhance its affinity for CME adaptor proteins, but the detailed mechanism remains to be investigated.

The dependency of CME on the interaction between cargo proteins provides a unique mechanism for the dynamic and tight coordination between the apical and lateral domain of tip growing cells. In some polarity systems, CME-based asymmetric removal of a membrane protein allows its polar distribution to the cortical domain where no CME occurs. For example, the asymmetric inhibition of CME is required for the polarization of PINs and Crumbs in *Arabidopsis* cells and *Drosophila* epithelial cells, respectively^62,66–68^. However, such CME-based mechanisms for polarity formation cannot explain how the apical domain is tightly and dynamically coordinated with the lateral/basal domain when the apical domain is moving and/or reorienting rapidly. In tip growing pollen tubes, ROP1 is activated locally in the extreme apex with lateral spreading through a diffusion-reaction positive feedback mechanism, while the REN1 RhoGAP-mediated global inhibition plays a major role in limiting the lateral spreading^18,23^. In contrast to REN1, REN4 acts as an important spatiotemporal rheostat in the fine-tuning of the apical ROP1 activity. When accumulating to a high level at the tip during the slow phase of growth, the active ROP1 interacts with REN4, inducing the CME-dependent removal of the REN4-ROP1 complex and preventing ROP1 over-accumulation at the tip. The REN4 rheostat is particularly important when ROP1 is over-activated at the tip, because in the absence of REN4, mild overexpression of ROP1 induced severe ROP1 over-activation and growth depolarization in pollen tubes. Our computational modeling reproduced the periodic and tightly coordinated redistribution of active ROP1 and REN4 that depends on their interaction with CME. Hence, by interacting with active ROP1 and mediating CME, REN4 acts as a spatiotemporal rheostat that fine tunes the level of apical ROP1 activity and dynamically generates a demarcation to coordinate the active ROP1-containing apical domain and the flanking REN4-containing lateral domains.

In fast tip-growing fungal hyphae, CME is also enriched at the boundary between apical and lateral domains and plays a role in the regulation of both polarity and growth of hyphae^42,43^. Hence CME may also regulate the dynamic coordination between the apical domain containing active CDC42 and the lateral domain. The role of the collar endocytosis triggered by cargos interaction in the spatiotemporal coordination of the apical and lateral polarity may be a common key feature for cells undergoing rapid tip growth.

Our findings also suggest that REN4 provide a noise-reducing mechanism for stabilizing pollen tube growth direction by fine-tuning the spatiotemporal distribution of active ROP1 via CME. This hypothesis is supported by several lines of evidence, including the *ren4-1* pollen tube phenotypes with wavier growth direction, the spatiotemporal distribution of REN4, and its physical and functional interaction with active ROP1. Loss of REN4- induced wiggly (zigzag) pollen tube shape in vitro (Fig. 1a, b), suggesting that REN4 plays an important role in stabilizing pollen tube growth direction. In WT pollen tubes, active ROP1 can transiently spread to the lateral PM domain, but is rapidly removed by the REN4-dependent endocytosis (Supplementary Movie 2). In the *ren4-1* pollen tubes, we observed wider and prolonged spreading of CRIB4-GFP to the lateral PM domain (Supplementary Fig. 4b, c and Supplementary Movie 5). Computational modeling shows that the unrestrained active ROP1 is prone to perturbation by noises, while REN4, by distributing to the two sides of the active ROP1 peak, resists the random relocation of the active ROP1 and stabilizes the growth polarity (Fig. 6b, c). By localizing to the lateral and collar domain, REN4 interacts with the expanded active ROP1 and triggers CME of the REN4-active ROP1 complex. Hence, REN4 has overlapping but distinct roles with REN1 in maintaining cell polarity of the apical active ROP1 signaling. The REN4-dependent CME quickly removes active ROP1 in the lateral PM to maintain the active ROP1 to the apical domain, thereby stabilizing the direction of tip growth. In contrast, the global inhibitor REN1 down-regulates the overall level of ROP1 activity to maintain polar tip growth of pollen tubes^23^.

Based on our findings here, we propose a working model for the REN4’s function as a rheostat in the spatiotemporal regulation of pollen tube tip growth (Fig. 6e). A diffusion reaction-based positive feedback mechanism induces the lateral proliferation of active ROP1 from the tip apex toward the lateral PM domain. At the collar region, the active ROP1 is counteracted by the lateral REN4 by their interaction, which triggers the CME-mediated removal of the ROP1/REN4 complex. We propose that if the level of the apical ROP1 activity reaches over a threshold level, the REN4/ROP1-mediated CME spreads to the apex to remove the REN4/ROP1 at the apex. The threshold level of the apical active ROP1 may be reached when the global inhibitor REN1 is saturated or when a defect in the REN1-dependent negative feedback mechanism (e.g., a defect in the polar exocytosis that targets REN1 to the tip) occurs^18^. This REN4-ROP1 encountering mechanism will not only dynamically maintain the demarcation between the apical and lateral domains, but will also dynamically fine tune the apical ROP1 activity to allow the regulation of this activity to a proper level. Consequently, the REN4/CME-based rheostat provides an important design principle for the robust control of tip growth direction, as well as the modulation of tip growth rates.

## Methods

### Plant and pollen tube growth

*Arabidopsis* (Columbia ecotype) plants were grown in growth rooms with 16 h photoperiod/8 h dark at 22 °C. The method of pollen tube germination is described in Hwang’s paper^23^. For cellular observation and measurement, mature *Arabidopsis* pollen grains from opened flowers were dusted onto the solid pollen germination medium (18% sucrose, 0.01% boric acid, 1 mM CaCl_2_, 1 mM Ca(NO_3_)_2_, 1 mM MgSO_4_, pH 6.4 and 0.5% agar). For Latrunclin B (LatB)-sensitized screens for pollen tube mutants, mature pollen grains were dusted on the solid pollen germination medium containing 0.8–1.0 nM LatB. After incubation at 28 °C for 1 or 3-4 h, frozen at −20 °C for 3 min to quickly terminate the PG and PTG, pollen tubes were observed under an inverted microscope (Nikon eclipse TE300) and images were taken pictures by use of a cooled CCD camera (Hamamatsu CA4742-95). Pollen tube lengths and tip widths were measured using Image J^69^. For CO-IP assay, mature pollens isolated from open flower were suspended in liquid pollen germination medium, and cultivated for 3 h for pollen tube collection.

### Plasmid DNA constructs

To generate entry clones, the *Arabidopsis* genomic DNA or flower cDNA and PrimeSTAR^®^GXL DNA Polymerase (Takara) were used to amplify specific REN4, EAP1, TML, CLC1 fragments for plasmid DNA constructs using specific primer listed in Supplementary Table 1. 2470 bp REN4 promoter fragment was amplified by the REN4 pro-F and REN4 pro-R; Full length of REN4 CDS with and without stop codon was amplified by REN4 CDS-F and REN4 CDS-R/REN4 CDS-RT; Full length REN4 genic DNA fragment including its upstream 2449 bp promoter and 1398 bp REN4 coding sequence with and without stop codon were amplified by using primers REN4 pro-F and REN4 CDS-R/REN4 CDS-RT; Full length EAP1 genic DNA with its upstream 827 bp was amplified from WT genomic DNA using following primers: EAP1-207F and EAP1-207R; Full length TML genic DNA with its upstream 769 bp was amplified using TML-207F and TML-207R; Full length CLC1 genic DNA with its upstream 271 bp CLC1 was amplified using CLC1-207F and CLC1-207R. REN4 N terminal for interactor screening by yeast two hybrid assay was amplified using REN4-CDS F and REN4-N-R from CDNA template. Full length ROP1 CDS with stop codon was amplified using primers ROP1-207F and ROP1-207R. Artificial microRNA (amiR)-based silencing approach was applied to investigate the function of REN4 in *Arabidopsis* pollen tube^70^. REN4 amiRNAs were designed according to the WDM (http://wmd3.weigelworld.org/cgi-bin/webapp.cgi) procedure. Six primers, pRS300-207A, pRS300-207B, REN4 I miR-s, REN4 II miR-a, REN4III miR*s, and REN4 IV miR*a were used to amplify specific fragments from the template of pRS300 vector (gift from the Detlef Weigel group). These amplified fragment was cloned into the vector of pENTR™/D-TOPO^®^ or pDONR™207 (Invitrogen) using the pENTR/D-TOPO cloning kit (Invitrogen, catalog number: K240020) or BP Clonase™ II Enzyme Mix (Invitrogen, catalog number:11789020). These fragments in pENTR™/D-TOPO^®^ or pDONR™207 were verified by sequencing. pDONR™207-ROP1 was mutated for the entry plasmid of pDONR207-CA-ROP1 using CA-ROP1(G15V)-F and CA-ROP1(G15V)-R and pDONR207-DN ROP1 using DN-ROP1 (D121A)-F and DN-ROP1(D121A)-R primers.

To generate expression clones, these fragments of interest were shuttled into the gateway destination vectors by the LR recombination reaction using LR Clonase II enzyme mix (Invitrogen, catalog number: 11791019). The entry clones of REN4, TML, and CLC1 without stop codon with their native promoter was shuttled into vector pGWB4 for fusion with GFP. The entry clones of REN4 and EAP1 without stop codon with their native promoter was shuttled into vector pGWB653 (ref.^71^) for protein fused with RFP. The entry clones of REN4 with native promoter and stop codon was combined with modified vector pCAMBIA3300:Lat52 pro:GFP-NOS: GW (Gateway cassette) for the generation of REN4 complementary and overexpression construct. The construction for REN4 promter activity assay was generated by LR reaction between the entry clones of REN4 promoter and vector pGWB3 (Invitorgen). The REN4 amiR’s was assemblied into a modified vector pCAMBIA3300:Lat52 pro: GW (Gateway cassette) for REN4 amiRNA silencing constructs. For construct of REN4 fused with MBP, the REN4 CDS with stop codon was shuttled into pDest-566 (Addgene). Entry clones of CA-ROP1 and DN-ROP1 with stop codon were used in a single Gateway LR reaction with pDest15 (Invitrogen) to generate N-terminal glutathione S-transferase (GST) fusion proteins.

For REN4 transient expression fused with RFP in tobacco pollen tube, the full length REN4 with stop codon was amplified using primers REN4-CDSF (EcoR1) and REN4-CDSRT (Spe1), the PCR product digested by EcoR1 and Spe1 was ligated into a modified vector PUC19: Lat52 pro: RFP (gift from Weihua Tang group, SIPPE, CAS) for N-terminal RFP fusion proteins.

### RT-PCR and GUS staining

Total RNA was extracted from roots, seedlings, stems, leaves, buds, opening flowers, and siliques of *Arabidopsis* using E.Z.N.A.^®^ Total RNA Kit I (Omega Bio-tek, catalog number:R6834-01). After treatment by DNAase (Promega, catalog number:M6101), 1 μg total RNA was transcribed into cDNA using ReverTra Ace-a-First StrandcDNA synthesis kit (Ferment, catalog number:FSK-101) according to the manufacturer’s instructions. The diluted cDNA product as template was used for mRNA transcript level analysis by RT-PCR and QRT-PCR using the specific primers. Ubiqutin10 mRNA transcript as control refer was amplified by UBQ10-F and UBQ10-R. GUS activity was visualized by staining the root, stem, leaf, inflorescences, pollen tube, and pistil from heterozygous pGWB3:: *REN4*pro::GUS transgenic lines, overnight in X-Gluc solution (0.5 mg/ml x-gluc, 0.1% TritonX-100, 2 mM K_4_Fe(CN)_6_·H_2_O, 2 mM K_3_Fe(CN)_6_, 100 mM sodium phosphate, 10 mM EDTA), and all tissues expect pollen tube were then cleared in 75% (v/v) ethanol. Finally, the GUS-stained tissue was mounted into the slide and photographed using microscope (Leica DM2500).

### Immunostaining of ROP1 protein in the pollen tubes

To visualize tip-localized ROP proteins in *Arabidopsis* pollen tubes, in vitro cultured pollen tubes were used for immunostaining against ROP1 antibody. Briefly *Arabidopsis* pollen tubes were fixed in the 4% paraformaldehyde solution [50 mM PIPES (pH 6.9), 3 mM MgSO_4_, 2 mM CaCl_2_, 18% sucrose and 4% paraformaldehyde] and then washed in PBS. The cell wall of washed pollen tubes was digested in the solution (2% cellulase R-10, 15 mM MES, pH 5.5, 400 mM Mannitol and 5 mM CaCl_2_) at room temperature for 5–10 min. Treated pollen tubes were incubated with the primary anti-ROP1 antibody (1:300) in the PBST solution for 4 h at RT or overnight at 4 °C after background blocking in the 1% nonfat milk for 1 h at RT. After washing with PBS containing 0.05% Triton X-100, slides were incubated with a secondary antibody (1:1000 FITC-conjugated goat anti-rabbit IgG, Sigma, catalog number: F9887) in PBST at RT for 2 h. After washing three times with PBST, slides were mounted with 0.1% p-phenylenediamine and 50% glycerol in PBS, and pollen tubes were observed on CLSM (Leica SP8).

### Transient expression in tobacco pollen tubes

*Nicotiana tabacum* plants were grown in growth chambers at 22 °C under a light regime of 12 h darkness and 12 h light, and mature pollen grains collected from these plants were used for transient expression using a particle bombardment procedure. Pollen grains suspended in a pollen germination medium (GM) containing 5 µM CaCl_2_, 5 µM Ca(NO_3_)_2_, 1 mM Mg(SO_4_)_2_, 0.01% H_3_BO_3_, and 18% sucrose, pH 6.5–7.0. A drop of 80–100 µl medium containing pollen grains from three flowers was applied to a piece of 2 × 2-cm nylon membrane (Micron Separations, Inc.) placed on top of a piece of 90-mm filter paper (Whatman) in a 100 × 15-mm Petri dish. Once liquid was drained from the membrane, an additional 1.5 ml GM was added to the filter paper. Pollen grains were then immediately bombarded with DNA-coated gold particles using a Biolistic PDS-1000/He particle delivery system (BioRad, catalog number: 165-2257). The microcarrier launch assembly was placed in the second slot from the top (level 2), and the target cells were placed at level 4 or 5 (the distance from the stopping screen to target cells is 8 or 11 cm, respectively). Rupture disks of 1100 psi were chosen. Firstly coating of 8 μl of 60 mg/ml gold particles (*d* = 1 μm) with 0.6 µg plasmid DNA of *Lat52* pro:RFP, *Lat52* pro:GFP: ROP1, *Lat52* pro:GFP and *Lat52* pro:RFP:REN4, then transfer these plasmids into the tobacco pollen using the PSD-1000/He particle delivery system (BioRad), respectively. After 2–3 h cultivation of bombarded pollen in the germination medium, the fluorescence positive pollen tubes were observed using confocal microscopy.

### Confocal microscopy and visualization of fluorescence protein

Localization patterns for fluorescence-tagged proteins in pollen tubes were observed using a confocal microscope (Leica SP8) and spinning disc confocal microscopy with a CSU-X1 spinning disc head (Yokogawa, Tokyo, Japan) equipped with a CFI Apo TIRF 1003 NA1.49 oil immersion objective and an Evolve EMCCD camera. Quantification of fluorescence intensities on the PM and pollen tube growth were done using the image J (Rasband, W.S. National Institute of Health) software packages. To simultaneously quantify the pollen tube growth and the fluorescence intensity of REN4, CRIB4, CLC1, a kymograph was produced from a line along the growth axis of the pollen tube using multiplekymograph plugin of Image J. Subsequent analyses were conducted on these kymographs. A background threshold, which was the background signal outside of the interested pollen tube, was set subsequently to define the peripheral region of pollen tube apex, and the intensity along pollen tube apex PM was measured according to the list of plot profile. The real-time sum of growth is measured by counting the position pixel between frames. In order to enhance the contrast, the pseudo-color “Fire” was set by Image J.

FRAP assay were performed using a confocal microscope (SP5), and the lateral region of REN4-GFP pollen tube was photo bleached using 100% power with 488-nm laser, and the recovery of fluorescence in pollen tube was monitored at 3-s intervals. The fluorescence recovery data obtained were corrected for bleaching during imaging as followed: the percentage of inherent photobleaching (PIP) for each frame during recovery was determined by measuring the change in fluorescence intensity of a region that was not initially photobleached. This fluorescence intensity in the initial bleached regions was multiplied by (1 + PIP) for each frame.

### Yeast two hybrid screen for REN4 interactors

Yeast two-hybrid screening was performed using the Matchmaker™ Gold Yeast Two-Hybrid System (Clontech). The cDNA library was generated using mRNA isolated from mature stamen and pistil in *Arabidopsis* open flowers. The bait construct (pGBKT7-REN4 (1-97 amino acid)) was generated and transformed into *Saccharomyces* *cerevisiae* strain Y2H gold strain, and test for autoactivation and toxicity. The two-hybrid library between the bait strain and library strain (Y187) was screened using the yeast mating method according to the manufacturer’s protocols. The screened colonies were analyzed on synthetically defined medium supplemented with X-α-Gal (SD/-Ade/-His/-Leu/-Trp/X-α-Gal medium). The blue colonies were selected for prey plasmid isolation. The prey plasmids were transformed into *S*. *cerevisiae* strain Y2HGold and used for autoactivation and toxicity assay. Then the prey and bait pGBK-REN4 plasmids were co-transformed into *S. cerevisiae* strain Y2HGold for verification of genuine interactions in yeast cells.

### Protein expression and purification

For protein expression in *E*. *coli*, REN4 was fused with MBP, and ROP1 was fused with GST. Fusion proteins were expressed at 16 °C for overnight after induction with 0.3 mM isopropyl-b-d-thiogalactopyranoside. *E*. *coli* cells were centrifuged at 5000 rpm for 10 min. For the purification of GST fusion proteins and MBP fusion proteins, cell pellets were resuspended in a binding buffer (20 mM Tris, pH 7.4, 200 mM NaCl, 1 mM EDTA, and 1 mM DTT) and sonicated using 15 s pulses for eight times. The supernatant was obtained by centrifugation at 12,000 rpm for 20 min. The supernatant was mixed with glutathione-agarose beads (Sigma-Aldrich, catalog number: G4510) or amylose beads (New England Biolabs, catalog number: E8021L) for GST fusion proteins and MBP fusion proteins, respectively. After 4 h of incubation, beads were washed with the binding buffer and eluted using maltose (10 mM) or glutathione (30 mM), respectively.

### **In vitro** protein interaction assays

Direct interaction between REN4 and ROP1 was investigated by in vitro pull down assays using *E. coli* expressed fusion proteins. REN4 fused with MBP and CAROP1/DN-ROP1 fused with GST were expressed in *E. coli* as described above. Approximately 1 μg of GST-ROP1 fusion proteins were incubated with GTP or GDP in binding buffer (50 mm Tris–HCl, pH 7.5, 10 mm MgCl_2_,1 mm DTT, 10 mg/ml bovine serum albumin, and 5 mm EDTA) containing 1 mM GTP or GDP for 30 min with shaking at 30 °C. GTP bond GST-ROP1 and GDP bond GST-ROP1 was mixed with 1 μg of purified MBP or MBP-REN4 fused protein in Amylose agarose beads in the binding buffer, and incubated at 4 °C for 1 h. The beads were washed with the binding buffer with 1% Triton x-100 for four times. The proteins associated with the amylose agarose beads were resuspended in 10 μl of SDS–PAGE loading buffer, separated on a 10% (w/v) PAGE–SDS gel by electrophoresis, and transferred to PVDF membranes. The MBP and GST-fusion proteins were then detected using mouse anti-MBP antibody (1:15,000, Abmart, catalog number: M40003M) anti-GST antibody (1:2000, Abmart, catalog number: M20007L) and the secondary antibody of goat anti-mouse (ab6789) conjugated to horseradish peroxidase (Abcam, Massachusetts, USA, 1:5000) was used. The Pierce™ ECL Western Blotting Substrate (Thermo fisher, catalog number:32106) and SuperSignal™ West Femto Maximum Sensitivity Substrate (Thermo fisher, catalog number: 34096) used for exposure.

### Co-immunoprecipation analysis

Pollen tube cultivated by hang-drop was collected from liquid germination medium, then pelleted down at 3200 g for 5 min to pool pollen tubes for CO-IP assay. Total protein was extracted from pollen tubes using the NEB buffer (20 mM HEPES (pH7.5), 40 mM KCl, 1 mM EDTA, 1% Triton X-100, 1 mM PMSF and the protease inhibitor cocktail). Total protein supernatants were incubated with GFP-trap (Chromotek, catalog number: gta-20) or RFP-trap agarose beads (Chromotek, catalog number: rta-20) for 3 h at 4 ℃ on a rocker. The beads conjugated protein sample was centrifuged and washed in the wash buffer (20 mM HEPES (pH7.5), 40 mM KCl, 1 mM EDTA, 1 mM PMSF and the protease inhibitor cocktail). The beads with the bound proteins were boiled in the 2× SDS–PAGE sample buffer, and analyzed by immunoblotting with purified anti-REN4 (1:15,000; ABclonal Biological Inc., China), anti-GADPH (1:2000; ABclonal Biological Inc., China, catalog number: AC001), anti-GFP (1:1000; Abcam, catalog number: ab6556) or anti-RFP antibody(1:1000; Chromotek, catalog number:5f8-100). Then, the secondary antibody of goat anti-mouse (ab6789) and goat anti-rat (ab97057) conjugated to horseradish peroxidase (Abcam, Massachusetts, USA, 1:5000) was used. Blotted membranes were washed thoroughly and visualized using chemiluminescence according to the manufacturer’s protocol (Thermo Scientific, Super Signal West Femto Kit, catalog number: 34095), and exposed to Bio-Max ML film in darkroom. The time of exposure can range from a few seconds to a few minutes depending on the signal intensity. The blots were cropped such that at least one marker position is present per panel.

### REN4 model formulation

The REN4 model is developed based on a previous model of ROP1-exocytosis polarization in pollen tube tip growth^18^.

The exocytosis rate, *E*_*x*_, as a function of the meridional position on the PM, *x*, and time *t*, is determined by the local concentration of active ROP1, *R*:

1 $$E_x = k_{E_x}R^\alpha .$$

Since both ROP1 and REN4 are required to induce endocytosis, the local endocytosis rate, *E*_n_, is described by

2 $$E_{\rm{n}} = k_{E_{\rm{n}}}{\rm min}(R,N),$$

where the local endocytosis rate is proportional to the minimum value of local ROP1 (*R*) and REN4 (*N*).

The spatiotemporal dynamics of active ROP1 is determined by four processes: ROP1 activation, deactivation, internalization, and lateral diffusion:

3 $$\frac{{\partial R}}{{\partial t}} = k_{\rm a}R_{\rm {in}}\left( {1 - \frac{R}{{R_{\rm {max}}}}} \right)\frac{{K_N}}{{N + K_N}} - k_{\rm d}R - E_nR + D\frac{{\partial ^2R}}{{\partial x^2}}.$$

The activation of ROP1 is proportional to the amount of inactive ROP1 (*R*_in_), inhibited by REN4, and saturated by the local ROP1 capacity (*R*_max_). The deactivation of ROP1 is proportional to the concentration of active ROP1. The internalization of active ROP1 into the cytoplasm is mediated by endocytosis.

The inactive form of ROP1 (*R*_in_) is maintained as a cytosolic pool by RhoGDIs, thus is expressed as a term independent of the membrane location:

4 $$R_{\rm {in}} = R_{\rm {total}} - {\int} {R{\rm{d}}x} .$$

The coefficients *k*_a_ and *k*_d_ are determined by the local activity of GEF and GAP, which are dependent on the exocytosis rate. In our hypothesis, the polarization of ROP1 is due to local activation and global inhibition, therefore, *k*_a_ and *k*_d_ are proportional to the local or global rate of exocytosis, respectively:

5 $$k_{\rm {a}} = k_{\rm {pf}}E_x,$$

6 $$k_{\rm {d}} = k_{{\rm nf}}k_{\rm {E}},$$

*k*_pf_ and *k*_nf_ are exocytosis-independent constants determined by the enzyme activity and expression levels of GEF and GAP, respectively.

The distribution of REN4 on the PM is affected by membrane association, disassociation, internalization and lateral diffusion:

7 $$\frac{{\partial N}}{{\partial t}} = k_{\rm {as}} - k_{\rm {dis}}N - k_{\rm {in}}E_{\rm{n}}N + D\frac{{\partial ^2N}}{{\partial x^2}}.$$

The initial REN4 concentration is 0 for the entire PM. The initial conditions of ROP1 is an arbitrary local perturbation at *x* = 0. No flux boundary conditions are imposed for both ROP1 and REN4. The definitions and values of the parameters are listed in Supplementary Table 2.

If the advection of proteins due to membrane flow is considered, the equations for ROP1 and REN4 distribution become

$$\frac{{\partial R}}{{\partial t}} 	 = k_{\rm {a}}R_{\rm {in}}\left( {1 - \frac{R}{{R_{\rm {max}}}}} \right)\frac{{K_N}}{{N + K_N}} - k_{\rm {d}}R - E_{\rm{n}}R + D\frac{{\partial ^2R}}{{\partial x^2}} - v\frac{{\partial R}}{{\partial x}}, \\ \frac{{\partial N}}{{\partial t}} 	= k_{\rm {as}} - k_{\rm {dis}}N - k_{\rm {in}}E_{\rm{n}}N + D\frac{{\partial ^2N}}{{\partial x^2}} - v\frac{{\partial N}}{{\partial x}}.$$

We assume that the flow rate of membrane, *v*, is proportional to the local exocytosis rate, since membrane flow in pollen tubes is caused by the insertion of new membrane via exocytosis:

$$v = \left\{ {v_0E_x,x > x_0\,0,x = x_0 - v_0E_x,x < x_0} \right\}$$

where *v*_0_ is a constant and *x*_0_ is the position where exocytosis peaks.

Parameter setting for the mathematical model is described in Supplementary Table 2.

### Data availability

Source data for Figs 1–7 and Supplementary Figs 1-8 have been provided in Supplementary Movies 1–6. All other data supporting the finding of this study are available from the corresponding author upon reasonable request.

## Electronic supplementary material

Supplementary Information

Description of Additional Supplementary Files

Supplementary Movie 1

Supplementary Movie 2

Supplementary Movie 3

Supplementary Movie 4

Supplementary Movie 5

Supplementary Movie 6

Supplementary Movie 7
