# Supplementary material for: The REN4 rheostat dynamically coordinates the apical and lateral domains of Arabidopsis pollen tubes
Source: Nat Commun. 2018 Jul 3;9:2573. doi: 10.1038/s41467-018-04838-w (PMC6030205; doi:10.1038/s41467-018-04838-w)
Supplement: Supplementary file 1 — Supplementary Information [file 41467_2018_4838_MOESM1_ESM.pdf]

SUPPLEMENTARY INFORMATION

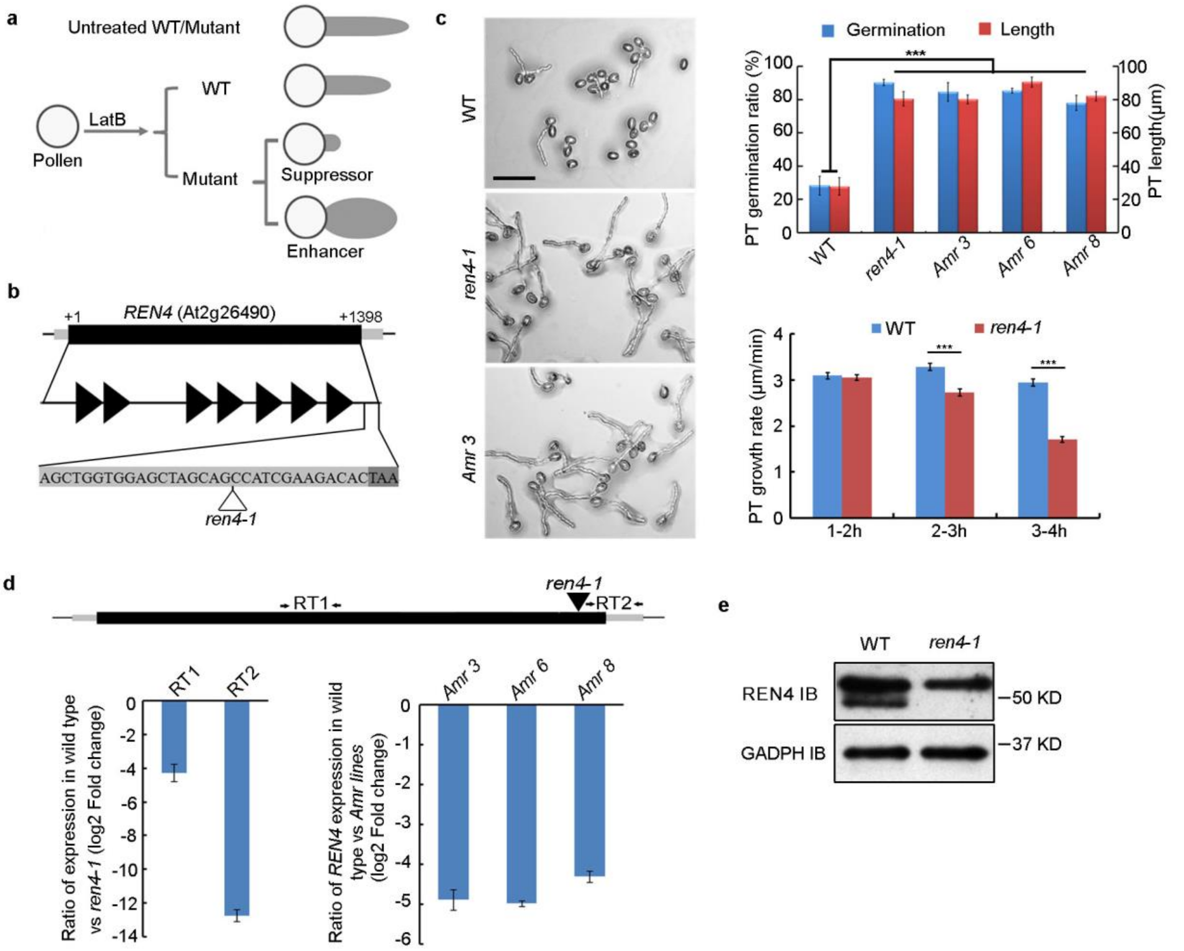

Supplementary Figure 1. ROP1 enhancer *ren4* shows defective in the pollen tube germination and growth, related to Figure 1

(a) A schematic of the LatB (Latrunculin B)-sensitive strategy for mutant screening of ROP1 positive and negative feedback regulator in the pollen tube. This strategy is based on the hypothesis that ROP1-dependent actin dynamic is involved in the feedback regulation of ROP1 activity for pollen tube tip growth. The pollen tubes of homozygote mutants form ABRC stock were analyzed in the pollen germination medium with low concentration LatB which don't affect the pollen tube polarity growth of wild type compared to that on the standard medium.

(b) The schematic representation of *REN4* gene structure, and the T-DNA insertion site (triangle) in *ren4* was denoted. Black box is the exon, black triangle is the motif of WD40, and the gray boxes are the UTR region.

(c) The pollen tube germination and elongation were promoted on medium after 1h cultivation, but the pollen tube growth rate of *ren4-1* is gradually slower compared to the wild type after 2h cultivation. The corresponding quantity measurement was shown. Data of pollen tube length are represented as mean ± SEM,*** p <0.001(One-way ANOVA, Turkey test), n ⩾ 100 from 3 biological replicates. Data of pollen tube germination are represented as mean ± SEM,*** p <0.001(two-tailed unpaired T-test), n ⩾15 from 3 biological replicates.Scale

bar=100 µm.

(d) The *REN4* expression has dramatically reduced in the pollen tubes of *ren4-1* mutant and Amr lines compared with wild type by QRT PCR analysis. Mean expression values are used after normalization with UBQ10 expression. The data are represented as mean± SEM from triple biological repeats.

(e) The REN4 is undetectable in the mutant pollen tube compared to the wild type using immunoblot assay of REN4 antibody, and GADPH as a loading control.

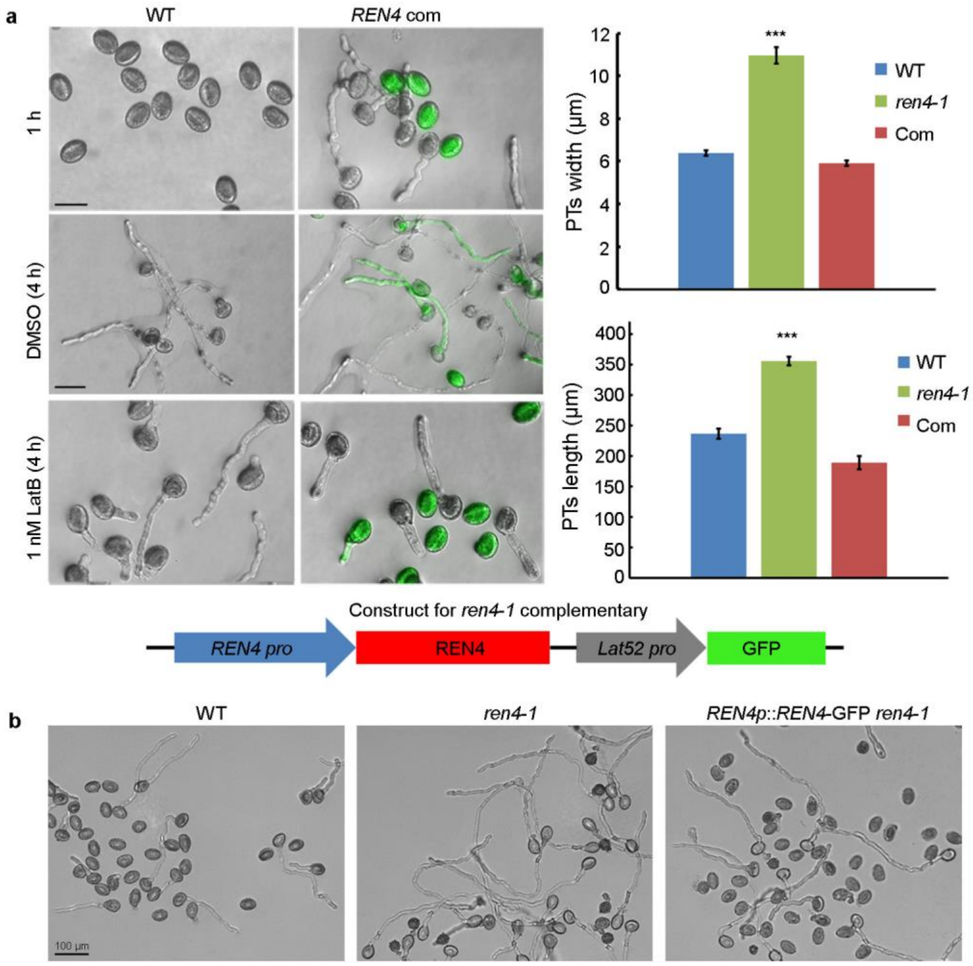

Supplementary Figure 2. REN4 expression recovered the mutant defective in pollen germination and tube growth, related to Figure 1

(a) The images and quantity analysis showed *ren4-1* pollen tube germination and 1nM LatB-induced the depolarized growth were recovered in the complementary transgenic line (T1 generation) of *REN4*pro-REN4/*Lat52*pro-GFP in Arabidopsis after 1 h and 4 h cultivation on the medium respectively. Data are represented as mean ± SEM, n ≥60 from 3 biologcial replicates , *** *p* <0.001 (two-tailed unpaired T-test) .

(b) The images showed that the expression of REN4 fused with GFP has recovered ren4-1 pollen and pollen tube growth defective.

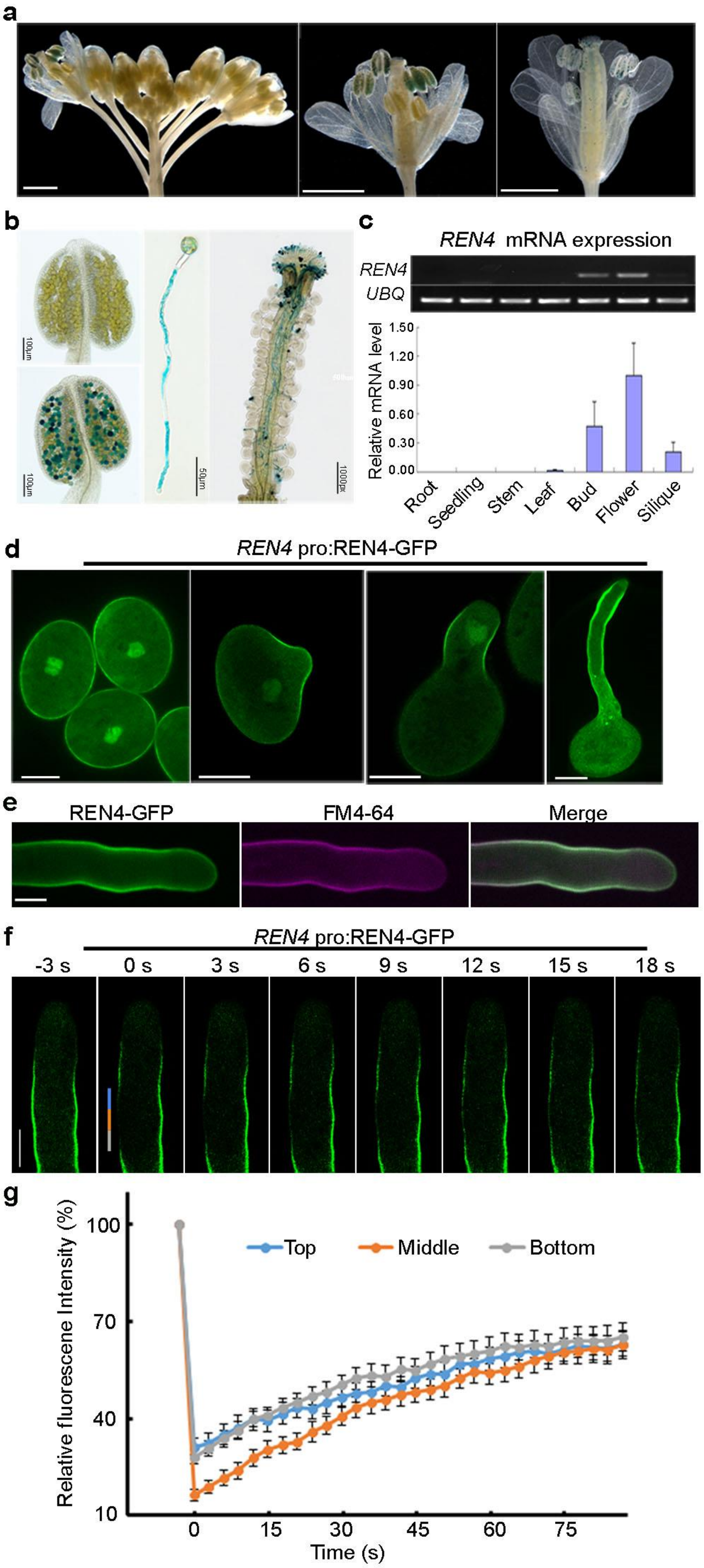

Supplementary Figure 3. The phylogenetic, transcription and protein localization analysis of REN4, related to Figure 1.

(a) The inflorescence with GUS stained stamen in the open flower of *REN4*pro: GUS transgenic line. Scale bars=500 μm.

(b) The GUS staining analysis of *REN4*pro: GUS mature pollens (up: unelongated anther, down: elongated anther) and pollen tubes growth *in vitro* and *in vivo*.
(c) The relative REN4 mRNA expression in the tissue of root, seedling, stem, leaf, bud, open flower and silique by the RT-PCR and qRT-PCR assay.
(d) Subcellular localization of REN4 in the mature pollen and germinated pollen tube of Arabidopsis. Scale bar=5 µm.
(e) REN4-GFP shows PM colocalization with FM4-64 dye in the Arabidopsis pollen tube. Scale bar=5 µm.
(f) A Time course analysis of REN4-GFP dynamic diffusion in the PM of a growing pollen tube using a FRAP assay. The initial bleached PM region was divided into three sub-regions, top, middle, and bottom, as indicated by blue, yellow, gray colors, respectively. Scale bar=5 µm.
(g) The fluorescence recovery curve of REN4-GFP in the bleached PM region of pollen tubes. The data shown are means from ten independent FRAP experiments. Error bars indicate SE.

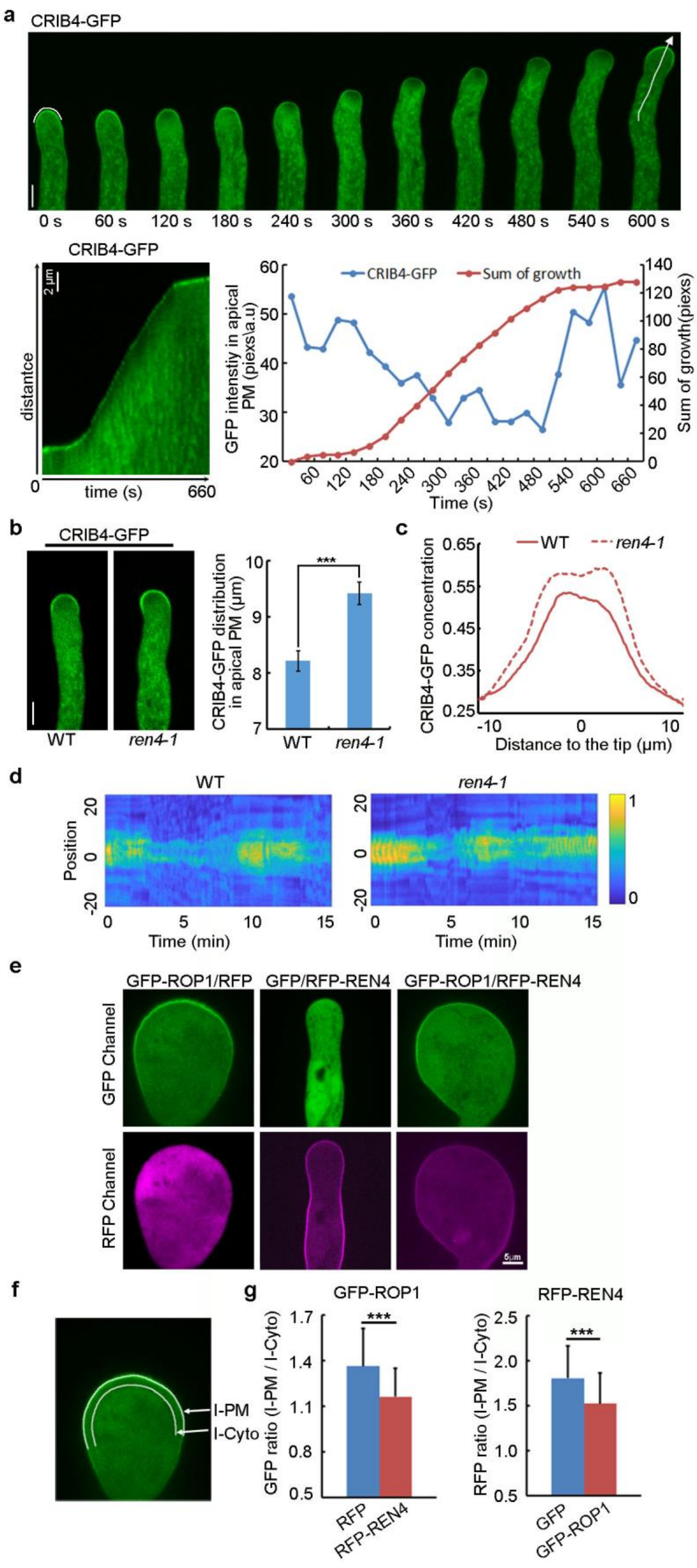

Supplementary Figure 4. The relationship between REN4 and active ROP1 in apical PM of pollen tubes, related to Figure 2

(a) A representative time series images and kymograph analysis of corresponding line which is along the tip center of *Lat52pro:CRIB4-GFP* pollen tube from the movie shown in Supplementary Movie 4 (n=65 frames, t=650 s). Scale bar=5 μm. Correlation between pollen tube net growth and CRIB4-GFP intensity at the apical PM was shown. The net tip growth was calculated by net advance of the cell tip margin between two consecutive images. CRIB4-GFP intensity is the average value of 9.5 μm apical PM using Image J (The region example is shown in 0 s image of E).
(b) The representive image (left) and measurement data (right) of CRIB4-GFP distribution in apical PM of wild type and *ren4-1* pollen tube. Data are represented as mean ±SEM, n⩾40 from 3 replicates, *** *p* <0.001(two-tailed unpaired T-test).
(c) The dynamic distribution of active ROP1in the apical PM of WT and *ren4-1* pollen tubes. The relative concentration of CRIB4-GFP (fluorescence intensity) along the 10 μm PM region on each side of the apical submit PM was measured from 5 independent movies (n=180 frames, t=900 s). Dashed red line shows that active ROP1 expanded in *ren4-1*.
(d) The dynamic distribution of CRIB4-GFP in apical PM of a representative WT and *ren4-1* pollen tube, respectively. The normalized GFP intensity along the 20 μm apical PM region on each side of the apical submit (indicated by 0) PTs was calculated and color-coded. See Videos S5 (n=180 frames, t=900 s) and S6 (n=180 frames, t=900 s).
(e) The PM distribution of GFP-ROP1 and RFP-REN4 in tobacco pollen tubes transiently co-expressing both of them (GFP-ROP1/ RFP-REN4), GFP-ROP1 and RFP, or RPF-REN4 and GFP. All assays were performed in the same experiment on the same batch of pollen. RFP-REN4 did not affect the polarity of GFP-ROP1 overexpressing tubes, this was likely due to slower assembly or activation of the REN4-RFP protein compared to GFP-ROP1 in tobacco pollen tubes transiently expressing these constructs.
(f) Schematic view of the measurements of the average GFP-ROP1 or RFP-REN4 intensity on the PM and in the cytosol.
(g) The change in GFP-ROP1 and RFP-REN4 localization indicated by the ratio of the average GFP in-tensity at the PM and in the cytosol caused by the transient co-expression of ROP1 and REN4 in tobacco pollen tube. For each measurement, n≥53 tubes from three biological replicates were used. Error bars indicate S.D.*** *p* <0.001(two-tailed unpaired T-test).

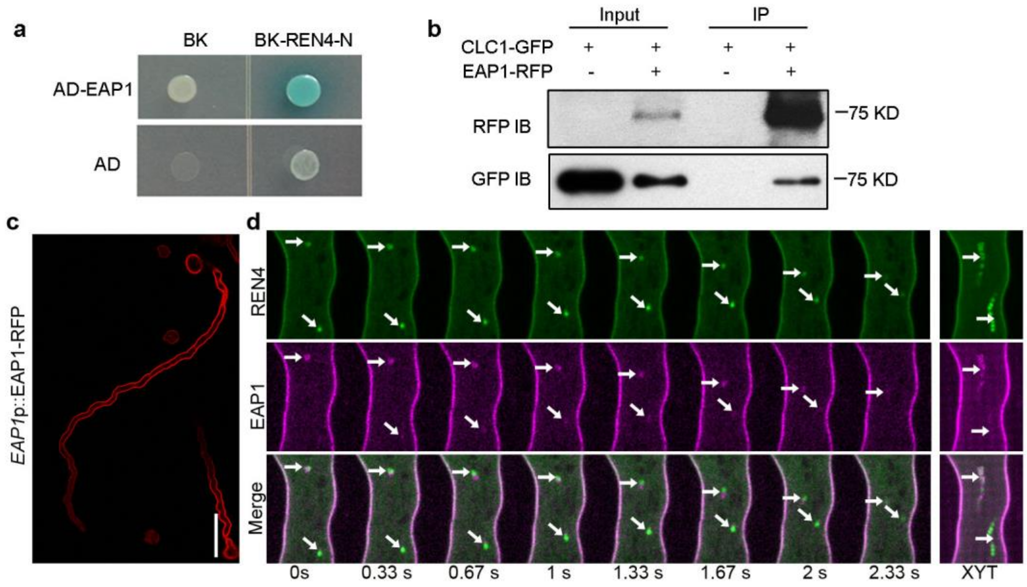

Supplementary Figure 5. EAP1 interacts with REN4 and regulates pollen tube growth, related to Figure 4

(a) The Y2H assay indicated the REN4 and EAP1 interaction.

(b) The co-immunoprecipitation showed the interaction between EAP1 and CLC1 in Arabidopsis pollen tube of *EAP1 pro*::*EAP1-RFP* transgenic line

(c) EAP1-RFP fused protein driven by *EAP1* native promoter is only localized in the PM and cytosolic along the whole pollen tube in Arabidopsis. Scale bar=50 µm.

(d) EAP1-RFP and REN4-GFP colocalizes in both PM and cytosolic in the pollen tube. Time series of a spinning disc movie (0.33s/frame) showing the dynamic behavior of REN4-GFP and EAP1-RFP. Arrow indicated representative foci in the cytosol.

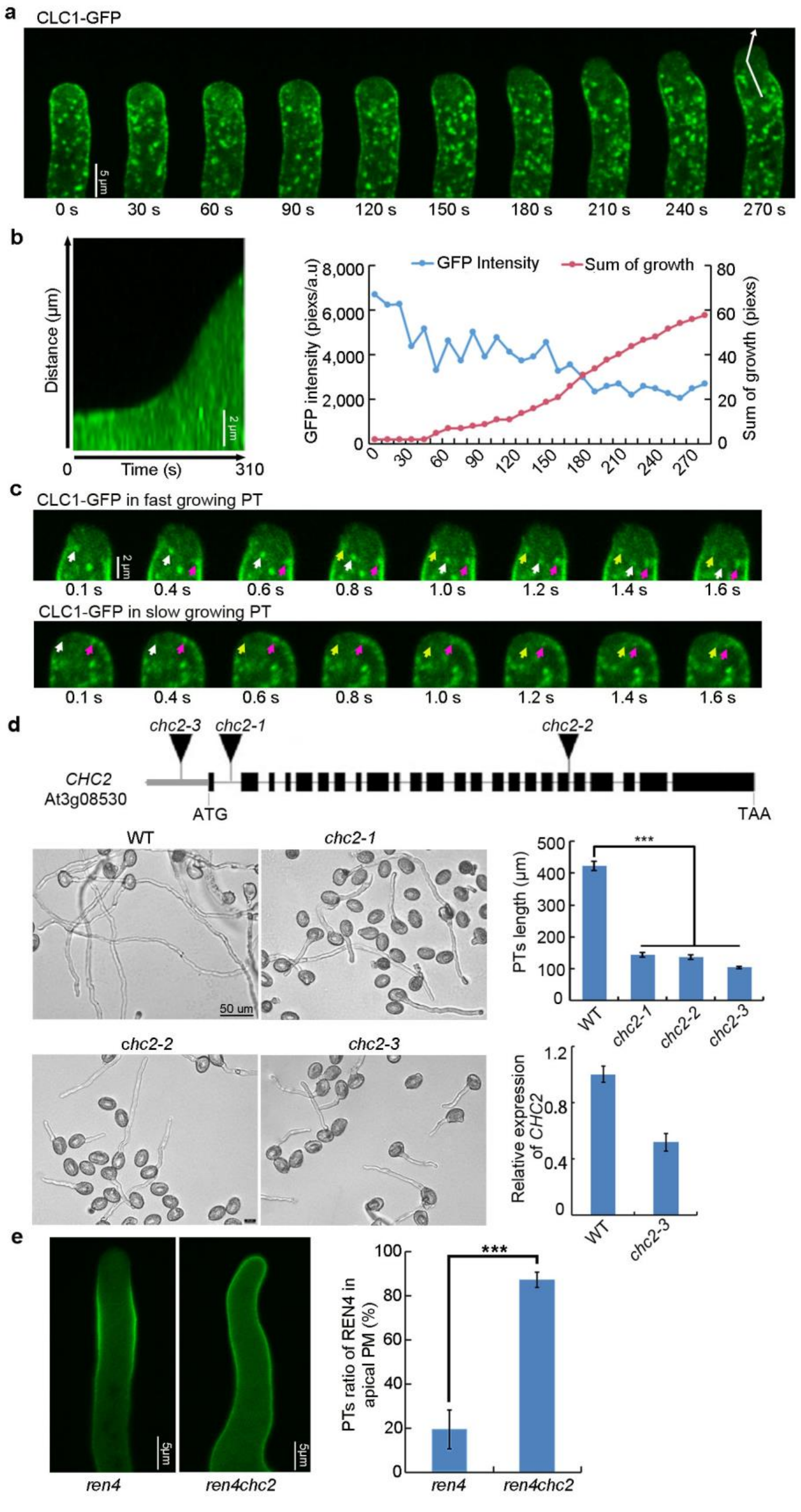

Supplementary Figure 6. The dynamic distribution and regulatory role of clathrin-mediated endocytosis in Arabidopsis pollen tube, related to Figure 5

(a) Time-lapse images of Clathrin-mediated endocytosis, labeled by CLC1-GFP, in a

wild-type (WT) pollen tube.

(b) Kymograph analysis of images shown in (A) (left) and a plot of the pollen tube growth and the amount of apical clathrin light chain versus time (right). The line for CLC1-GFP kymograph is along the pollen tube apical center shown in image of time series. The mean GFP intensity of the 9.5 μ m apical PM of PTs was calculated and the net tip growth was measured by net advance of the cell margin between two consecutive images.

(c) Time-lapse images of CLC1-GFP labeled vesiculars internalization from PM into cytosol in fast and slow growth pollen tube in Arabidopsis. The time series from a representative movie (Movie S6 and S7) (n = 16 frames, t = 1.6 s). Arrows indicate CLC-GFP particles were internalized into the cytoplasm from the PM. Scale bar=2 μm. See also Movie S6 and S7.

(d) The pollen tubes of wild type and three alleles of *chc2* mutant after 3h cultivation on the pollen germination medium. T-DNA insertion site were denoted in the schematic gene structure of *CHC2*. Black box is the exon, black triangle is T-NDA insertion site, the gray boxes indicated the promoter region, and the gray lines represented the introns. Data are represented as mean ± SE, $n \geqslant 67$ from 3 biological replicates, *** $p < 0.001$(One-way ANOVA Tukey-test). And the CHC2 mRNA transcription level was compared between wild type and *chc2-3* by qRT-PCR.

(e) Representative images of REN4-GFP distribution in the pollen tube of *ren4-1* and *ren4-1chc2* mutant. Right is the measurement data of pollen tube ratio with REN4-GFP in apical PM.

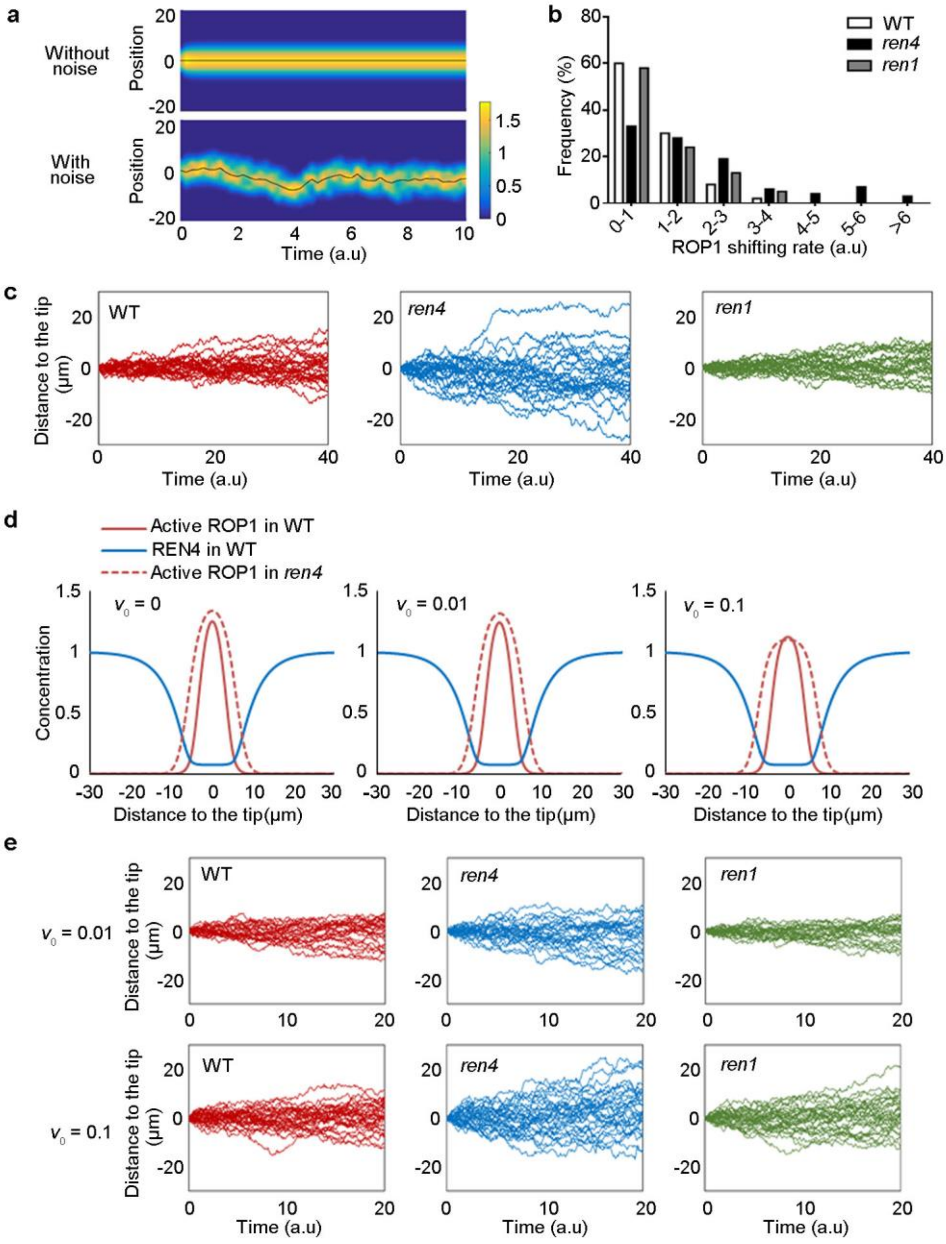

Supplementary Figure 7. REN4 as a lateral inhibitor stabilizes the distribution of active ROP1, related to Figure 6

(a) Simulated distribution of active ROP1 over time without (upper panel) or with (lower panel) noise in the activation rate of ROP1. Color shows the concentration of active ROP1. Black solid lines indicate the center of ROP1 distribution.

(b) Histogram of the distribution of ROP1 shifting rate in each genotype.

(c) Simulated distribution of active ROP1 over time in the presence of noise. Each line represents an individual trace of the center of ROP1 distribution, which shows significantly higher variance in *ren4* than in WT or *ren1*.

(d) and (e) Simulations of modified model considering the advection of membrane proteins. (d) Simulated distribution of active ROP1 and REN4 when v_0 is 0 (no advection), 0.01 (low

advection) or 0.1 (high advection). Dashed red line shows that active ROP1 expands in the absence of REN4. (e) Simulated distribution of active ROP1 over time in the presence of noise when v_0 is 0.01 or 0.1 (n = 25). Each line represents an individual trace of the center of ROP1 distribution, which shows significantly higher variance in *ren4* than in WT or *ren1*.

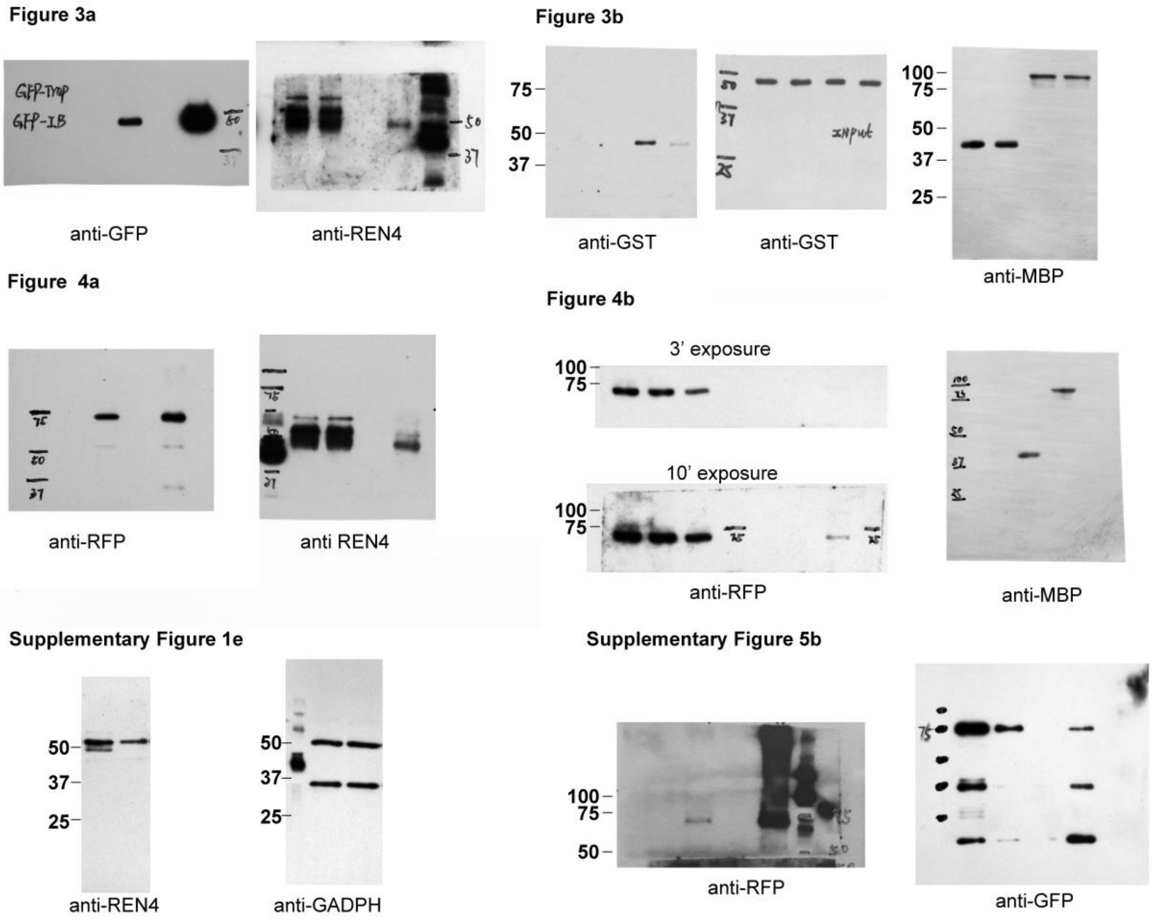

Supplenetary Figure 8. Original images for immunoblots bands in Figures 3a, 3b, 4a, 4b, Supplementary Figures 1e and 5b were shown. The molecular weight markers and the antibodies used are indicated on the images.

Supplementary Table 1: List of Primers Used, Related to the Method Section

Oligo name	Sequence
REN4 Pro-F	5'cacctggtgagaacgaaattacgt 3'
REN4 pro-R	5'gctatcagcttccgtaaacat 3'
REN4 CDS-F	5'caccatgtttacggaagctgatag 3'
REN4 CDSN-R	5' accttcttcacggacaagc 3'
REN4 CDS-R (no stop)	5'gtgtcttcgatggctgctag 3'
REN4 CDS-RT (stop)	5'ttagtgtcttcgatggctgc 3'
EAP1-207F	5'ggggacaagtttgtacaaaaaagcaggctgcgcttgggttttgttgttttga 3'
EAP1-207R	5'ggggaccactttgtacaagaaagctgggtcgaatgtaatcaaatctggcatt 3'
TML-207F	5'ggggacaagtttgtacaaaaaagcaggctgcacagaatggacattttgtacaaacac 3'
TML-207R	5'ggggaccactttgtacaagaaagctgggtcattgcacatatagactccggt 3'
CLC1-207F	5'ggggacaagtttgtacaaaaaagcaggctgctgtcgtctctttccgttaaag 3'
CLC1-207R	5'ggggaccactttgtacaagaaagctgggtcctccgccttggttccctcggc 3'
PRS300-207A	5'ggggacaagtttgtacaaaaaagcaggctgcctgcaaggcgattaagttgggtaac 3'
PRS300-207B	5'ggggaccactttgtacaagaaagctgggtcgcggataacaatttcacacaggaaacag 3'
REN4 I miR-s	5'gatatgttggcatattgtgcccttctctcttttgtattcc 3'
REN4 II miR-a	5'gaagggcacaatatgccaacatatcaaagagaatcaatga 3'
REN4 III miR*s	5'gaagagcacaatatggcaacatttcacaggtcgtgatatg 3'
REN4 IV miR*a	5'gaaatgttgccatattgtgct cttctacatatatattcct 3'
ROP1-207F	5'ggggacaagtttgtacaaaaaagcaggctgcatgagcgcttcgaggttcgt 3'
ROP1-207R	5'ggggaccactttgtacaagaaagctgggtctcatagaatggagcatgcctt 3'
CA-ROP1(G15V)-F	5' gacggttggtgatgtagctgtcggaaaaac 3'
CA-ROP1(G15V)-R	5' gtttttccgacagctacatcaccaaccgtc 3'
DN-ROP1(D121A)-F	5' gttggaacaaagcttgctcttcgagatgataaac 3'
DN-ROP1(D121A)-R	5' gtttatcatctcgaagagcaagctttgttccaac 3'
UBQ10-F	5'cacttggtcctcaggctcc 3'
UBQ10-R	5'cagcaagagttctgccatcc 3'
REN4 RT1-F	5'aagaaacaccgcactgctct 3'
REN4 RT1-R	5'tcatcatgagctgggattga 3'
REN4 RT2-F	5'gcagccatcgaagacactaa 3'
REN4 RT2-R	5'aaaaattagttcaaactcacctcca 3'
salk_018094-LP	5' ctgaacacacgtttctcatcg 3'
salk_018094-RP	5' gtcacagctttggctgtaagc 3'
salk_028826-LP	5' aaaagtcatgacacttcttccattc 3'
salk_028826-RP	5' aattcgaggaaaccgttatgg 3'
salk_042321-LP	5' tgttctgcaagttcatgttcg 3'
salk_042321-RP	5' aggtggatgacctggaagaag 3'
salk_049520-LP	5' tttcatggttagctccaccac 3'

salk_049520-RP	5' acggtcgattccaggagatac 3'
CHC2-QRT-F	5' gaaggccaaagagcaggaag 3'
CHC2-QRT-R	5' atatggtggcatgggaggc 3'
REN4-F(*E*coRI)	5' aaagaattccgatgtttacggaagctgatag 3'
REN4-RT(*S*peI)	5' aaaactagtttagtgtcttcgatggctgcta 3'

Supplemental Table 2. Parameter setting for the mathematical model

Variables	Definition	Unit
x	Meridional position on the PM	μm
t	Time	s
R	The concentration of active ROP1 in the PM	1
N	The concentration of REN4 in the PM	1
R_{in}	The concentration of inactive ROP1 in the cytoplasm	1
k_{a}	ROP1 activation coefficient	1 s^{-1}
k_{d}	ROP1 deactivation coefficient	1 s^{-1}
E_{x}	Exocytosis rate	1
E_{n}	Endocytosis rate	1
**Constants**	**Definition**	**Value**
$R_{\max}$	Maximum active ROP1 concentration on the PM	5
R_{total}	Total ROP1 (including active and inactive ROP1)	30
D	Diffusion coefficient of ROP1 and REN4 on the PM	0.2 μm^2 s^{-1}
k_{Ex}	Coefficient linking ROP1 activity with exocytosis rate	1
k_{En}	Coefficient linking the interaction of ROP1-REN4 with endocytosis rate	1.7
α	Coefficient representing the degree of nonlinearity in the positive feedback	1.25
k_{pf}	Strength of exocytosis-mediated positive feedback	0.035 s^{-1}
k_{nf}	Strength of exocytosis-mediated negative feedback	0.2 s^{-1}
k_{as}	Rate of REN4 association with the PM	0.01 s^{-1}
k_{dis}	Rate of REN4 disassociation with the PM	0.01 s^{-1}

k_{in}	Coefficient of endocytosis-mediated internalization of REN4	1
K_N	Half inhibition rate of ROP1 activation by REN4	0.8
