# Supplementary material for: The REN4 rheostat dynamically coordinates the apical and lateral domains of Arabidopsis pollen tubes
Source: Nat Commun. 2018 Jul 3;9:2573. doi: 10.1038/s41467-018-04838-w (PMC6030205; doi:10.1038/s41467-018-04838-w)
Supplement: Supplementary file 2 — Description of Additional Supplementary Files [file 41467_2018_4838_MOESM2_ESM.pdf]

Description of Additional Supplementary Files:

Supplementary Movie 1: **REN4 distribution in the pollen tube**, related to Fig. 1. Showing the REN4 dynamic localization in plasma membrane of pollen tube tip region from fast growing stage to slow growing stage in the Arabidopsis pollen tube of *REN4* pro:REN4-GFP complimentary line. Scale bars=5um, numbers show time were placed (min:sec).

Supplementary Movie 2: **The distribution of active ROP1 and REN4 in the pollen tube**, related to Fig. 2. The dynamic distribution of CRIB4-GFP (active ROP1 marker) and REN4-RFP was shown at the apical PM of Arabidopsis pollen tube with expressing of CRIB4-GFP and REN4-RFP. Scale bars=5um, numbers show time were placed (min:sec).

Supplementary Movie 3: **The distribution of clathrin-mediated endocytosis and REN4 in the pollen tube**, related to Fig. 4. The dynamic distribution of CLC1-GFP and REN4-RFP was shown at the apical PM of Arabidopsis pollen tube with expressing of CLC1-GFP and REN4-RFP. Arrow indicated the vesicular internalization site of clathrin-mediated endocytosis. Scale bars=5um, numbers show time were placed (min:sec).

Supplementary Movie 4: **The distribution of active ROP1 in the WT pollen tube**, related to Supplementary Figure 4. The dynamic distribution of active ROP1 marker (CRIB4-GFP) at the apical PM of Arabidopsis WT pollen tube was shown. Scale bars=5um, numbers show time were placed (min:sec).

Supplementary Movie 5: **The distribution of active ROP1 in the *ren4-1* pollen tube**, related to Supplementary Figure 4. The dynamic distribution of active ROP1 marker (CRIB4-GFP) at the apical PM of Arabidopsis the *ren4-1* pollen tube was shown. Scale bars=5um, numbers show time were placed (min:sec).

Supplementary Movie 6: **The daynamic of clathrin-mediated endocytosis in the fast growing pollen tube**, related to Supplementary Figure 5C. CLC1-GFP labeled vesicular internalization and trafficking in the fast growing pollen tube of Arabidopsis was shown.

Supplementary Movie 7: **The daynamic of clathrin-mediated endocytosis in the fast growing pollen tube**, related to Supplementary Figure 5C. The CLC1-GFP labeled vesicular internalization and trafficking in the slow growing pollen tube of Arabidopsis was shown.
